# Ultrafast Brain MRI at 3 T for MS: Evaluation of a 51-Second Deep Learning-Enhanced T2-EPI-FLAIR Sequence

**DOI:** 10.3390/diagnostics14171841

**Published:** 2024-08-23

**Authors:** Martin Schuhholz, Christer Ruff, Eva Bürkle, Thorsten Feiweier, Bryan Clifford, Markus Kowarik, Benjamin Bender

**Affiliations:** 1 Department of Diagnostic and Interventional Neuroradiology, Eberhard Karls University, University Hospital, 72076 Tübingen, Germany; christer.ruff@med.uni-tuebingen.de (C.R.); eva.buerkle@med.uni-tuebingen.de (E.B.); benjamin.bender@med.uni-tuebingen.de (B.B.); 2 Siemens Healthineers AG, 91052 Erlangen, Germany; thorsten.feiweier@siemens-healthineers.com; 3 Siemens Medical Solutions USA, Boston, MA 02478, USA; bryan.clifford@siemens-healthineers.com; 4 Department of Neurology and Stroke, Neurological Clinic, Eberhard Karls University, University Hospital, 72076 Tübingen, Germany; markus.kowarik@med.uni-tuebingen.de

**Keywords:** ultrafast brain MRI, multi-shot EPI, ultrafast FLAIR, deep learning, image acceleration, image enhancement, multiple sclerosis, inflammatory brain lesions

## Abstract

In neuroimaging, there is no equivalent alternative to magnetic resonance imaging (MRI). However, image acquisitions are generally time-consuming, which may limit utilization in some cases, e.g., in patients who cannot remain motionless for long or suffer from claustrophobia, or in the event of extensive waiting times. For multiple sclerosis (MS) patients, MRI plays a major role in drug therapy decision-making. The purpose of this study was to evaluate whether an ultrafast, T2-weighted (T2w), deep learning-enhanced (DL), echo-planar-imaging-based (EPI) fluid-attenuated inversion recovery (FLAIR) sequence (FLAIR_UF_) that has targeted neurological emergencies so far might even be an option to detect MS lesions of the brain compared to conventional FLAIR sequences. Therefore, 17 MS patients were enrolled prospectively in this exploratory study. Standard MRI protocols and ultrafast acquisitions were conducted at 3 tesla (T), including three-dimensional (3D)-FLAIR, turbo/fast spin-echo (TSE)-FLAIR, and FLAIR_UF_. Inflammatory lesions were grouped by size and location. Lesion conspicuity and image quality were rated on an ordinal five-point Likert scale, and lesion detection rates were calculated. Statistical analyses were performed to compare results. Altogether, 568 different lesions were found. Data indicated no significant differences in lesion detection (sensitivity and positive predictive value [PPV]) between FLAIR_UF_ and axially reconstructed 3D-FLAIR (lesion size ≥3 mm × ≥2 mm) and no differences in sensitivity between FLAIR_UF_ and TSE-FLAIR (lesion size ≥3 mm total). Lesion conspicuity in FLAIR_UF_ was similar in all brain regions except for superior conspicuity in the occipital lobe and inferior conspicuity in the central brain regions. Further findings include location-dependent limitations of signal-to-noise ratio (SNR) and contrast-to-noise ratio (CNR) as well as artifacts such as spatial distortions in FLAIR_UF_. In conclusion, FLAIR_UF_ could potentially be an expedient alternative to conventional methods for brain imaging in MS patients since the acquisition can be performed in a fraction of time while maintaining good image quality.

## 1. Introduction

Echo-planar imaging (EPI) represents a very fast acquisition technique in magnetic resonance imaging (MRI). It was first described by Mansfield in 1977 [1] and has since been refined. In [2], Poustchi-Amin et al. reviewed the main principles of EPI: to reduce the number of excitation pulses, thus reducing the number of repetition time (TR) periods by acquiring multiple k-space lines per shot, i.e., during a repetition period. It can be performed using only one shot (single-shot EPI), which means that k-space is filled completely following one excitation, or otherwise using multiple shots (multi-shot EPI), i.e., a particular fraction (obeying a regular sampling pattern along the phase-encoding direction) of k-space is filled with data within each TR period. This can be achieved by generating repetitive echo train signals. Apart from diffusion-weighted imaging (DWI), perfusion-weighted imaging (PWI), functional MRI (fMRI), and occasionally T2*-weighted (T2*w) imaging [3], EPI has not been integrated into other routine brain MRI sequences such as fluid-attenuated inversion recovery (FLAIR) for the assessment of inflammatory lesions due to drawbacks in terms of image quality and artifacts.

Multiple sclerosis (MS) is an inflammatory disorder of the central nervous system (CNS). Neuro-inflammation thereby results in demyelination and axonal damage with reactive gliosis and lesion formation leading to clinical disability progression [4]. MS is the most frequent demyelinating disease worldwide, with the highest prevalence levels in Europe and North America (over 100/100,000 inhabitants) [5]. Relapsing–remitting multiple sclerosis (RRMS) is the most frequent initial course of MS, and women are affected two to three times (or more) as often as men [5]. According to the McDonald criteria and its later revisions, dissemination of CNS lesions in time and space needs to be fulfilled for diagnosis, thus inevitably requiring appropriate MRI examinations [6,7,8,9]. Again, the diagnosis of MS entails further scans for disease activity and treatment monitoring [10]. For this purpose, a FLAIR sequence is an essential tool for cerebral imaging, and the availability of a fast imaging technique would be relevant in that context, given the high prevalence of this disease and the corresponding costs.

In 1999, Filippi et al. analyzed MS lesion detectability in ultrafast EPI-FLAIR images compared to conventional fast spin-echo FLAIR images. They observed similar lesion numbers for lesions that were greater than ten millimeters in long-axis diameter. Detection of smaller lesions proved inferior using EPI-FLAIR sequences [11]. Owing to specificity reasons, there has been support for a size threshold of white matter lesions to allow the diagnosis of MS [12,13]. According to the McDonald criteria, hyperintense areas are referred to as lesions if they are greater than three millimeters in long axis [6,7,8,9]. Grahl et al. recently reviewed those stipulations about a lesion size threshold and confirmed three millimeters to be a reasonable threshold to account for diagnostic criteria in MS for three-dimensional (3D) MRI acquisitions at 3 tesla (T) [14]. However, the effectiveness of EPI-FLAIR sequences has been far from meeting those targets and has therefore not been a suitable option for routine MRI scans.

In recent years, the evolution of artificial intelligence-based applications has witnessed a substantial surge, and significant advancements in machine learning have gained great attention in the field of medical imaging, opening up unprecedented opportunities [15]. Applied to image reconstruction, these deep learning (DL) techniques provide an improved trade-off between speed, resolution, and signal-to-noise ratio (SNR), and often enable significant reductions in scan times when combined with (highly) accelerated conventional techniques such as parallel imaging (PI) [16,17,18,19]. Alternative methods include compressed sensing (CS) [20,21,22,23], simultaneous multi-slice (SMS) imaging (also known as multiband imaging) [24,25,26,27,28], iterative denoising (ID) [29,30] and synthetic MRI [31,32,33,34,35,36,37,38].

Enabled primarily by DL, the acceleration provided by these techniques has redoubled interest in further investigations. Particularly, the integration of several of these methods with EPI-based imaging has led to the development of ultrafast multi-contrast protocols, providing all contrasts required in an emergency setting (T1, T2, T2*, T2-FLAIR, DWI) [39,40,41,42,43,44,45,46]. There is also an approach to incorporate various contrasts in one sequence [47,48,49,50,51,52]. So far, these methods have not targeted clinical applications that rely to some extent on high-resolution data. Also, current research that focuses on EPI-FLAIR in particular is limited and primarily addresses stroke patients and pediatric patients [53,54,55,56]. In-depth evaluations of MS patient brain images that were acquired using DL-enhanced EPI-FLAIR acquisition techniques have not been conducted to date to our knowledge. Although 3D-FLAIR is clearly given preference over 2D-FLAIR for diagnosis and monitoring of MS [57,58], the purpose of this part of our study was to evaluate whether a 2D ultrafast DL-enhanced EPI-FLAIR (FLAIR_UF_) sequence of the brain could be an adequate alternative to conventional FLAIR scans.

## 2. Materials and Methods

### 2.1. Study Design

**Figure 1:**
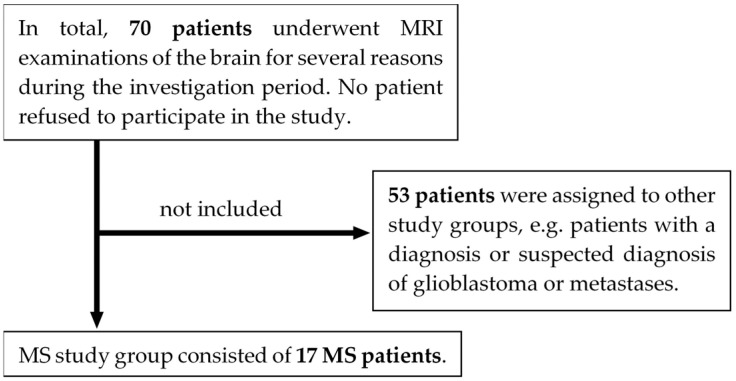
Flowchart of study participants. Note. MRI = magnetic resonance imaging; MS = multiple sclerosis.

This prospective, exploratory study was approved by the institutional review board (Project ID 031/2021BO2). We adhered to the 1964 Declaration of Helsinki and its later amendments. Inclusion criteria were adulthood (≥18 years), a routine MRI examination to be carried out on the 3 T MAGNETOM Vida (Siemens Healthineers AG, Forchheim, Germany) scanner, and at least three of the following five MRI contrasts to be conducted: T1-weighted (T1w), T2w, T2*w, DWI, or T2w FLAIR. Moreover, a criterion for allocation to the MS study group was having MS according to the McDonald criteria (2017) [9]. Exclusion criteria were lack of capacity to consent, missing written informed consent, acute stroke in the lysis time window, or general MRI contraindications, such as non-MRI-compliant implants or severe claustrophobia. As can be seen in Figure 1, 17 MS patients underwent 3 T MRI scans of the brain between May 2021 and March 2022. For details on patient characteristics, see Table 1. All eligible patients gave written informed consent to participate in this voluntary study.

### 2.2. Imaging Protocol and Image Acquisition

All examinations were conducted using a 3 T MRI scanner (see Section 2.1). We used a 20-channel head and neck coil and applied our standard protocol for patients with MS, incorporating the following contrasts: 3D T1 magnetization prepared rapid gradient echo (MPRAGE), contrast-enhanced 3D T1-MPRAGE, contrast-enhanced axial T1 turbo/fast spin echo (TSE), infratentorial axial T2-TSE, axial DWI, 3D double inversion recovery (DIR), and 3D-T2-FLAIR (FLAIR_3D_). In three patients, an axial standard T2-TSE-FLAIR (FLAIR_TSE_) sequence was added as well. Additionally, we acquired the ultrafast axial T2w FLAIR_UF_ research sequence in all patients, along with other ultrafast sequences that are not in the scope of this article (i.e., two native axial T1w contrasts, two contrast-enhanced axial T1w contrasts, axial DWI, and an axial sequence providing a T2*w and a T2w contrast). All native sequences were acquired prior to the contrast agent application, and the standard protocol sequences were acquired in advance of the ultrafast sequences. The acquisition parameters for all three T2w FLAIR sequences (i.e., FLAIR_UF_, FLAIR_TSE_, and FLAIR_3D_) are given in Table 2. By using the FLAIR_UF_ sequence, which takes 0:51 min, the acquisition time can be reduced to almost a sixth of the time required for the FLAIR_3D_ sequence (4:57 min) and nearly a third of the time required for the FLAIR_TSE_ sequence (2:22 min).

After acquisition of the data and extraction of relevant clinical data, imaging data were de-identified using RSNA Clinical Trial Processor (CTP) software (RSNA CTP Java Version 1.8, Radiological Society of North America, Oak Brook, IL, USA) for further evaluation within this trial.

### 2.3. FLAIR_UF_ Sequence

The FLAIR_UF_ sequence examined in this study was a T2w inversion-recovery double-shot spin-echo EPI sequence; i.e., k-space data for each image were acquired in two shots with interleaved phase-encoding patterns. Each shot consisted of a spin-echo inversion recovery excitation module followed by a 64-echo readout train. In all three FLAIR sequences (FLAIR_UF_, FLAIR_TSE_, and FLAIR_3D_), the PI acceleration factor was two.

In order to alleviate the deterioration in image quality caused by the above-mentioned acceleration techniques, the FLAIR_UF_ sequence has several novel features. One of them is a DL-enhanced processing technique that employs a machine learning-based reconstruction to decrease image noise and residual aliasing [43,44,45,59]. Specifically, the method utilizes an unrolled gradient descent network to produce a high-SNR prior image, which is then used to regularize a final conjugate-gradient SENSE reconstruction (see [43] for further details). The network was trained on data acquired with a 20-channel head matrix coil at 3 T [43,44]. However, the clinical patient images included in our study were not part of the training data. The reconstruction method requires coil sensitivity maps based on the eigenvector-based iterative self-consistent parallel imaging reconstruction technique (ESPIRiT) [60]. Those maps are calculated using fast low-angle shot (FLASH)-based autocalibration scans acquired prior to the slice scans. Furthermore, geometric coil compression was utilized to improve reconstruction performance, reducing reconstruction times [61]. Another novel feature implemented is magnetization transfer (MT) preparation to improve tissue contrast, as described in [62]. In addition, the FLAIR_UF_ sequence utilizes the following techniques to improve image quality, amongst others: field map-based geometric distortion correction [63,64], phase correction scans to mitigate residual Nyquist ghosting, flow attenuation gradients to weaken signals from flowing or pulsating fluids, and automated interleaving of inversion and acquisition modules to further optimize the scan efficiency.

### 2.4. Image Evaluation

Inflammatory lesions and image quality were evaluated by one reader (M.S.), and all ratings were verified by an experienced neuroradiologist (B.B.) in a consensus reading with the first reader for a final decision.

#### 2.4.1. Lesion Assessment

**Figure 2:**
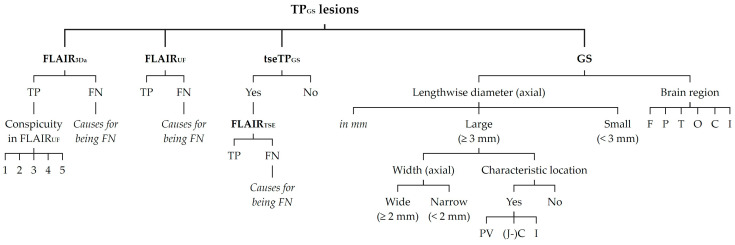
Classification of TP_GS_ lesions. Note. TP_GS_ lesions = total number of true-positive lesions detected using all contrasts available (gold standard); FLAIR_3Da_ = axial reconstruction of FLAIR_3D_; FLAIR_UF_ = ultrafast axial FLAIR; tseTP_GS_ = subset of TP_GS_ recorded with FLAIR_TSE_; FLAIR_TSE_ = axial standard TSE-FLAIR; GS = utilization of all contrasts available, particularly T2-FLAIR, T1, and T2; TP = true-positives; FN = false-negatives; 1 = better/larger in the FLAIR_UF_ images compared to FLAIR_3Da_; 2 = equal compared to FLAIR_3Da_; 3 = better in the FLAIR_3Da_ images, but classified as a lesion using only the FLAIR_UF_ images; 4 = better in the FLAIR_3Da_ images and classified as no lesion using only the FLAIR_UF_ images; 5 = FLAIR_3Da_ lesion that is not at all visible in the FLAIR_UF_ images; PV = periventricular; (J-)C = (juxta-)cortical; I = infratentorial (brainstem, cerebellum); F = frontal; P = parietal; T = temporal; O = occipital; C = central (insular lobe, corpus callosum, basal nuclei, diencephalon).

All inflammatory lesions recorded were documented and listed. Identifiers were assigned, using all sequence contrasts available, particularly axial reconstructions including 3D multiplanar reconstructions (MPRs) of FLAIR_3D_, DIR, and T1-MPRAGE, plus an axial T2-TSE sequence contrast. The utilization of all these contrasts was referred to as the gold standard (GS). Accordingly, the total amount of lesion counts was referred to as true-positives using GS (TP_GS_). To each TP_GS_ lesion, the following attributes were assigned (see Figure 2): (1) clearly detectable as a lesion using only the axial reconstruction of FLAIR_3D_ (FLAIR_3Da_), i.e., true-positive in FLAIR_3Da_ (TP_3Da_), or not clearly detectable as a lesion using only FLAIR_3Da_, i.e., false-negative in FLAIR_3Da_ (FN_3Da_); (2) clearly detectable as a lesion using only the FLAIR_UF_ images (TP_UF_), or not clearly detectable as a lesion using only the FLAIR_UF_ images (FN_UF_); (3) lesion size, i.e., lengthwise axial diameter (mm, to the nearest tenth); (4) lesion size category, i.e., lengthwise axial diameter (large [≥3 mm] or small [<3 mm]); (5) location, i.e., brain region (frontal, parietal, temporal, occipital, central [insular lobe, corpus callosum, basal nuclei, diencephalon], or infratentorial [brainstem, cerebellum]).

The following attributes were assigned to only a particular subset of the TP_GS_ lesions (see also Figure 2): (1) lesion width (wide [≥2 mm] or narrow [<2 mm]; axial) and lesion location, according to Barkhof et al. and the McDonald criteria [6,7,8,9,13,65] (characteristic [periventricular, juxta-/cortical, infratentorial] or not characteristic) were both assigned to large (≥3 mm) TP_GS_ lesions only; (2) lesion detectability in FLAIR_TSE_ (TP_TSE_ or FN_TSE_) was assigned to the subset of TP_GS_ recorded with FLAIR_TSE_ (tseTP_GS_); (3) lesion conspicuity in FLAIR_UF_ compared with FLAIR_3Da_ counterpart, using an ordinal five-point Likert scale (1 = better/larger in the FLAIR_UF_ images; 2 = equal; 3 = better in the FLAIR_3Da_ images, but classified as a lesion using only the FLAIR_UF_ images; 4 = better in the FLAIR_3Da_ images and classified as no lesion using only the FLAIR_UF_ images; 5 = FLAIR_3Da_ lesion that is not at all visible in the FLAIR_UF_ images), was only assigned to the TP_3Da_ lesions; (4) presumed causes for not being detected in FLAIR_UF_ were only assigned to the FN_UF_ lesions; (5) presumed causes for not being detected in FLAIR_3Da_ were only assigned to the FN_3Da_ lesions; (6) presumed causes for not being detected in FLAIR_TSE_ were only assigned to the FN_TSE_ lesions.

**Figure 3:**
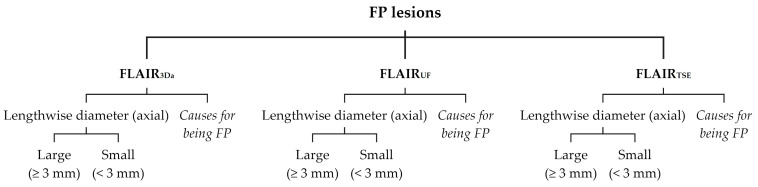
Classification of FP lesions. Note. FP = false-positive; further abbreviations as in Figure 2.

False-positive (FP) lesions were recorded and listed separately for FLAIR_UF_ (FP_UF_), FLAIR_3Da_ (FP_3Da_), and FLAIR_TSE_ (FP_TSE_). To each FP lesion, the following attributes were assigned (see Figure 3): (1) size category, i.e., lengthwise axial diameter (large [≥3 mm] or small [<3 mm]); (2) presumed causes for being mistaken for a lesion.

#### 2.4.2. Image Quality Assessment

For the purpose of general image quality assessment, three parameters were employed: signal-to-noise ratio (SNR), contrast-to-noise ratio (CNR), and artifacts. FLAIR_UF_, FLAIR_3Da_, and FLAIR_TSE_ slice series were each rated for SNR and CNR on an ordinal five-point Likert scale (1 = very good; 2 = good; 3 = acceptable; 4 = mediocre but diagnostic; 5 = poor and non-diagnostic). Moreover, all artifacts in the FLAIR_UF_, FLAIR_3Da_, and FLAIR_TSE_ images were assessed based on quantity and quality. To this end, FLAIR_UF_, FLAIR_3Da_, and FLAIR_TSE_ sequences were classified as to their limitations of diagnostic information in the respective artifact region, using an ordinal five-point Likert scale (0 = No artifact; 1 = Artifact exists, but diagnostic information is not limited; 2 = Artifact exists, and diagnostic information is slightly limited in the artifact region; 3 = Artifact exists, and diagnostic information is limited in the artifact region; 4 = Artifact exists, and diagnostic information is severely limited in the artifact region).

To assess location-dependent SNR and CNR within the FLAIR_UF_ images, the following attributes were assigned to each TP_GS_ lesion (see previous Section): (1) SNR in the vicinity of the lesion (standard or substandard; with reference to the average SNR in FLAIR_UF_); (2) CNR in the vicinity of the lesion (standard or substandard; with reference to the average CNR in FLAIR_UF_).

### 2.5. Statistical Analysis

Statistical analyses were performed post hoc using SPSS (IBM SPSS Statistics Version 29.0.0.0, IBM Corp., Armonk, NY, USA) and Excel (Microsoft Excel for Microsoft 365 MSO Version 2305, Microsoft Corp., Redmond, WA, USA).

In order to compare lesion detection in FLAIR_UF_ with lesion detection in FLAIR_3Da_, contingency tables were created, correlating TP and FN lesion counts. Also, the counts and presumed causes of FN and FP lesions were contrasted. The sensitivity values (TP/[TP + FN]) and positive predictive values (PPVs; TP/[TP + FP]) as to lesion detection were specified including their 95% Clopper–Pearson confidence intervals (CIs), and FN lesion counts were compared using McNemar’s test (Excel). The same analyses were performed to compare the lesion detection in FLAIR_UF_ with the lesion detection in FLAIR_TSE_. To ascertain factors that affect the lesion conspicuity within the FLAIR_UF_ images, conspicuity ratings were compared, and grouped by lesion size and location, using the Wilcoxon rank-sum test and the Kruskal–Wallis test (SPSS). With the aim to investigate whether conspicuity ratings might possibly have been biased by lesion size variations among the location groups, lengthwise diameters of the TP_3Da_ lesions were reported for each location (mean/95% CI, standard deviation [SD], median) and compared using the Kruskal–Wallis test (SPSS). Moreover, large TP_3Da_ lesions (≥3 mm) for each brain region were divided into wide (≥2 mm) and narrow (<2 mm) lesions and were further differentiated by conspicuity ratings.

For the purpose of contrasting the ordinal SNR, CNR, and artifact ratings in the FLAIR_UF_ and FLAIR_3Da_ image series, results were reported as median plus interquartile range (IQR) as well as mean ± SD; a Wilcoxon signed-rank test was also performed (SPSS). Similarly, results were reported for comparing FLAIR_UF_ with the FLAIR_TSE_ image series. To ascertain positional factors that affect the SNR and CNR within the FLAIR_UF_ images, the dichotomous SNR and CNR ratings were grouped by location; the proportions of the substandard ratings were determined for each group including 95% Clopper–Pearson CIs. Groups were compared using the chi-squared test (Excel).

In [66], Bender et al. stated that multiple testing corrections are not necessarily required for exploratory trials generating (diverse) hypotheses. Accordingly, adjustment for multiple comparisons was waived for this study. A *p*-value < 0.05 was considered statistically significant.

## 3. Results

### 3.1. Image Acquisitions and Lesions

**Figure 4:**
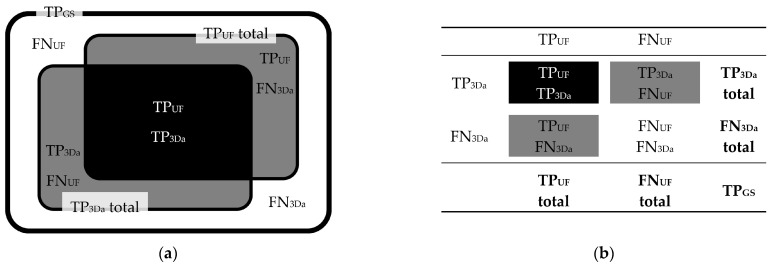
Correlations between TP_UF_, FN_UF_, TP_3Da_, FN_3Da_, and TP_GS_ lesion counts. Schematic illustration (**a**) and contingency table (**b**). Note. TP_GS_ = number of true-positive lesions detected using all contrasts available (gold standard); TP_UF_ = true-positive lesions detected in the FLAIR_UF_ images; FN_UF_ = false-negative lesions using the FLAIR_UF_ images; TP_3Da_ = true-positive lesions detected in the FLAIR_3Da_ images; FN_3Da_ = false-negative lesions using the FLAIR_3Da_ images.

**Figure 5:**
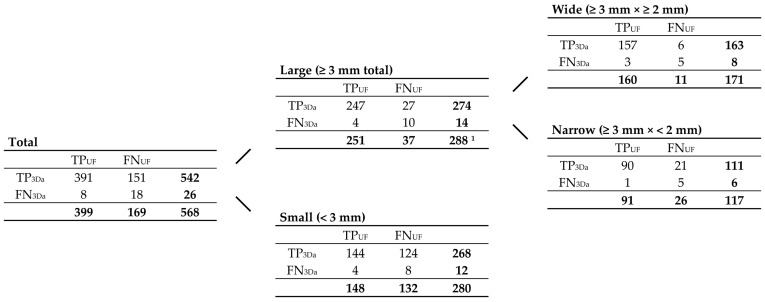
Contingency tables correlating TP_UF_, FN_UF_, TP_3Da_, FN_3Da_, and TP_GS_ lesion counts, grouped by size, according to Figure 4. Note. Abbreviations as in Figure 4. ^1^ 191 of them were ‘characteristic MS lesions’, including 109 periventricular lesions, 44 infratentorial lesions, and 38 (juxta-)cortical lesions.

The image data include seventeen FLAIR_UF_ sequences, seventeen corresponding FLAIR_3D_ sequences, and three additional FLAIR_TSE_ sequences, which could all be acquired from seventeen MRI examinations in sixteen different RRMS patients (twelve males, five females). The patients’ mean age was 29 years, with ages ranging from 21 to 60 years (see Table 1). Lesion counts are schematically visualized in Figures 4 and 8 and listed in detail in Figures 5 and 9. In total, we counted 568 true-positive lesions using the gold standard (TP_GS_), i.e., actual inflammatory brain lesions detected using all contrasts available, as described in Section 2.4.1. Of the 568 existing lesions, 288 (50.7%) were categorized as ‘large lesions’ (≥3 mm total), and 280 (49.3%) were classed as ‘small lesions’ (<3 mm). Of the 288 large lesions, 171 (59.4%) were named as ‘wide lesions’ (width ≥2 mm), and 117 (40.6%) were labeled ‘narrow lesions’ (width < 2 mm). Taking account of all 568 lesions, there were 542 (95.4%) of them that could be detected using only the FLAIR_3Da_ images, hence referred to as TP_3Da_ lesions. Of the 542 TP_3Da_ lesions, 274 (50.6%) were large and 268 (49.4%) were small. The count of wide large TP_3Da_ lesions was 163 (59.5%), and the count of narrow large TP_3Da_ lesions was 111 (40.5%). The mean count of TP_GS_ lesions and standard deviation per patient was 33 ± 21, with counts ranging from 1 to 87. Taking account of all 288 large TP_GS_ lesions, there were 191 (66.3%) lesions grouped as ‘characteristic MS lesions’, which were subdivided into three groups: 109 (57.1%) periventricular lesions, 44 (23.0%) infratentorial lesions, and 38 (19.9%) (juxta-)cortical lesions. All lesion counts, including TP_UF_ lesions and all corresponding FN lesion counts, correlated with each other, are considered in the following Section.

### 3.2. Lesion Detection

#### 3.2.1. FLAIR_UF_ Compared with FLAIR_3Da_

Correlations between TP_UF_, FN_UF_, TP_3Da_, FN_3Da_, and TP_GS_ lesions are schematically demonstrated in Figure 4. Lesion counts are specified in Figure 5, classified by size (large, small, wide, narrow). All counts of total TP and total FN lesions, as well as FP lesions, in both the FLAIR_UF_ images and FLAIR_3Da_ images, were analyzed, compared, and grouped by size.

**Figure 6:**
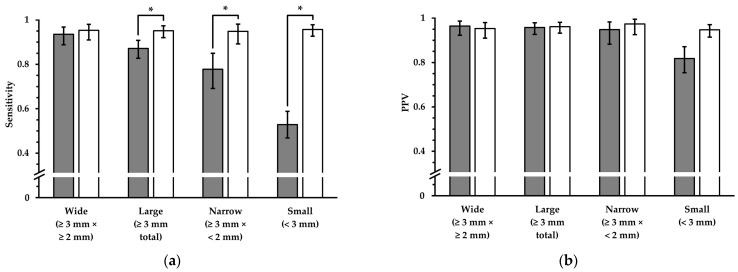
Sensitivity and PPV in terms of lesion detection, using FLAIR_UF_ images (gray) and FLAIR_3Da_ images (white). Four groups, which represent different lesion diameters, are displayed, respectively. The additional error bars denote the 95% CIs: (**a**) For wide lesions, no significant difference in sensitivity could be found. For the groups that comprise smaller lesions, however, the sensitivity was significantly inferior using the FLAIR_UF_ images, decreasing more and more as a function of lesion diameter. (**b**) No significant differences in PPV were found for any of the large lesion groups (large, wide, and narrow). For small lesions, the PPV in the FLAIR_UF_ images was moderately lower compared to the FLAIR_3Da_ group (no overlap between confidence intervals). Note. Abbreviations as in Table 3. * *p* < 0.05.

The sensitivity and PPV in terms of lesion detection using FLAIR_UF_ compared with FLAIR_3Da_ are analyzed in Table 3 and visualized in Figure 6. For wide lesions, no statistically significant differences could be found in either sensitivity (*p* = 0.50) or PPV (overlap between confidence intervals). Neither was there a significant difference in PPV for narrow lesions (overlap between confidence intervals). Nevertheless, the sensitivity was significantly inferior in the FLAIR_UF_ images for narrow large lesions (*p* < 0.001). For small lesions, the PPV was reduced in the FLAIR_UF_ images compared with the FLAIR_3Da_ images (no overlap between confidence intervals), and the sensitivity was considerably lower in the FLAIR_UF_ images compared with the FLAIR_3Da_ images (*p* < 0.001).

**Figure 7:**
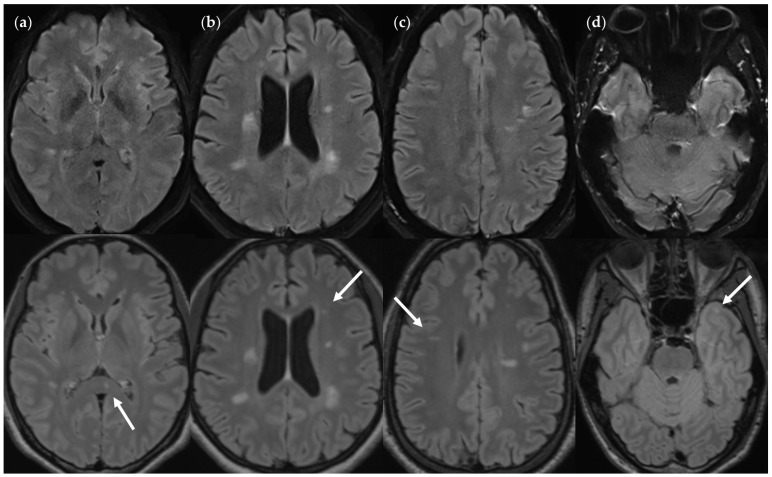
FN_UF_ lesions (top row) contrasted with their TP_3Da_ counterparts (arrow, bottom row). The images also show several other lesions: (**a**) Lesion in the splenium of the corpus callosum (approx. 3 mm × 2 mm). Causes for it not being detected may be image noise and slice thickness/slice gaps. (**b**) Left frontal lesion (approx. 3 mm × 1 mm). Causes may be associated with slice thickness/contrast as well as image noise. (**c**) Thin right frontal lesion (approx. 7 mm × 1 mm). It was mistaken for cortex in the FLAIR_UF_ image. (**d**) Left temporopolar lesion (approx. 3 mm × 2 mm). It was not recognized as such in the FLAIR_UF_ image owing to commonly occurring distortions within this region. Note. Corresponding slices could not be positioned exactly identically for two reasons: Different slice thicknesses including slice gaps (**c**) and non-parallel slice inclinations (**d**).

Presumed causes of FN lesions in both image variants are quantified in Table 4 (large) and Table 5 (small). Presumed causes for lesions not being detected using FLAIR_UF_ were (1) lesions were not visible owing to a combination of insufficient levels of spatial resolution, CNR, and SNR; (2) lesions were mistaken for cortex or other physiological structures owing to a combination of insufficient levels of spatial resolution, CNR, and SNR; and (3) lesions were concealed by distortion artifacts. Similarly, presumed causes for lesions not being detected using FLAIR_3Da_ were (1) lesions were mistaken for or masked by (mostly infratentorial) pulsation artifacts; (2) lesions were mistaken for cortex or other physiological structures; and (3) lesions were not visible at all, likely due to low resolution-related lesion contrast. Image examples of FN_UF_ lesions and corresponding TP_3Da_ lesions are given in Figure 7 and Figure 13c. Images of a FN_3Da_ lesion including the corresponding TP_UF_ lesion are shown in Figure 15b.

Frequent causes of FP lesions were in each case: partially imaged cortex or infratentorial pulsation artifacts appearing like lesions. While the former could be observed more frequently than the latter in the FLAIR_UF_ images, they occurred at a similar rate in the FLAIR_3Da_ images. Comprehensive results are reported in Table 6. Image examples of FP_UF_ and FP_3Da_ lesions are given in Figures 15 and 16e.

#### 3.2.2. FLAIR_UF_ Compared with FLAIR_TSE_

**Figure 8:**
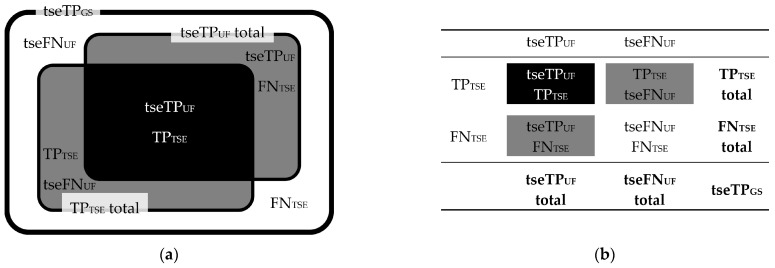
Correlations between tseTP_UF_, tseFN_UF_, TP_TSE_, FN_TSE_, and tseTP_GS_ lesion counts. Schematic illustration (**a**) and contingency table (**b**). Note. TP_GS_ = number of true-positive lesions detected using all contrasts available (gold standard); tseTP_GS_ = subset of TP_GS_ recorded with FLAIR_TSE_; tseTP_UF_ = corresponding subset of TP_UF_ recorded with FLAIR_TSE_; tseFN_UF_ = corresponding subset of FN_UF_ recorded with FLAIR_TSE_; TP_TSE_ = true-positive lesions detected in the FLAIR_TSE_ images; FN_TSE_ = false-negative lesions using the FLAIR_TSE_ images; further abbreviations as in Figure 4.

**Figure 9:**
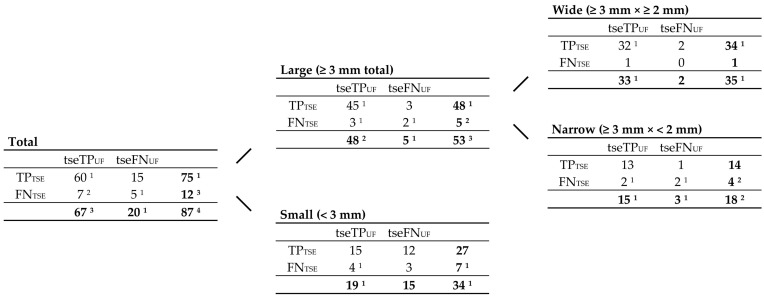
Contingency tables including a subset of those TP_GS_ lesions covered by the FLAIR_TSE_ sequence (tseTP_GS_). tseTP_UF_, tseFN_UF_, TP_TSE_, FN_TSE_, and tseTP_GS_ lesion counts are correlated, and grouped by size, according to Figure 8. Note. Abbreviations as in Figure 8. ^1^ One was FN using FLAIR_3Da_; the rest were TP in FLAIR_3Da_. ^2^ Two were FN using FLAIR_3Da_; the rest were TP in FLAIR_3Da_. ^3^ Three were FN using FLAIR_3Da_; the rest were TP in FLAIR_3Da_. ^4^ Four were FN using FLAIR_3Da_; the rest were TP in FLAIR_3Da_. Lesion counts without superscripts were all TP in FLAIR_3Da_.

**Figure 10:**
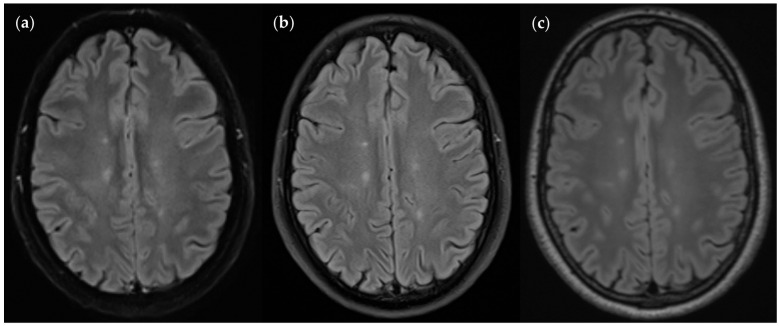
An MS patient received an MRI scan including three T2w FLAIR sequences: FLAIR_UF_ (**a**), FLAIR_TSE_ (**b**), and FLAIR_3D_ (**c**). Five lesions can be seen in each picture, situated in the frontoparietal region.

To compare FLAIR_UF_ with FLAIR_TSE_ images, all FLAIR_TSE_ data available were utilized and matched with their FLAIR_UF_ counterparts. TP_TSE_ and FN_TSE_ lesion counts added up to the corresponding subset of TP_GS_ lesion counts (tseTP_GS_). Again, tseTP_GS_ could be divided into corresponding subsets of TP_UF_ and FN_UF_ lesions each (tseTP_UF_ and tseFN_UF_). Correlations between tseTP_UF_, tseFN_UF_, TP_TSE_, FN_TSE_, and tseTP_GS_ lesions are schematically demonstrated in Figure 8. Lesion counts are given in Figure 9, classified by size (large, small, wide, narrow). Image examples of TP lesions in FLAIR_UF_, FLAIR_TSE_, and FLAIR_3Da_ are shown side by side in Figure 10. FP_TSE_ lesion counts along with corresponding tseFP_UF_ counts can be found in Table 10.

**Figure 11:**
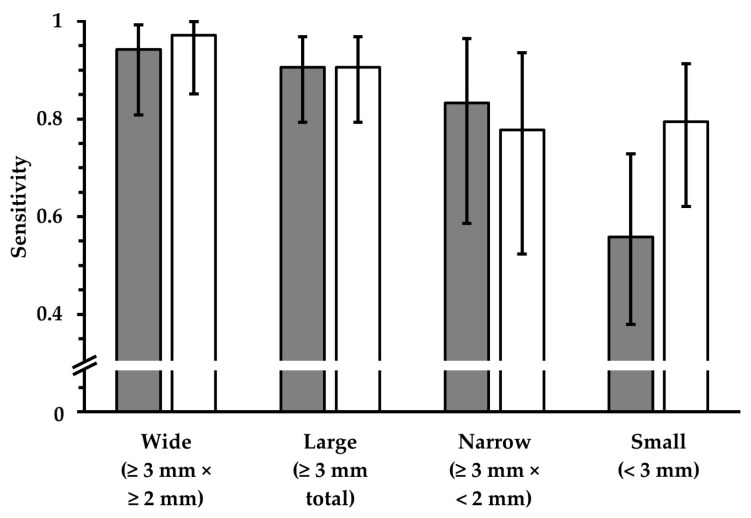
Sensitivity in terms of lesion detection, using FLAIR_TSE_ images (white) and corresponding FLAIR_UF_ images (gray). Four groups, which represent different lesion diameters, are displayed, respectively. The additional error bars denote the 95% CIs. No significant differences in sensitivity could be detected for any of the lesion groups (*p* > 0.05). For small lesions, however, the data suggest a lower sensitivity using the FLAIR_UF_ images compared to the FLAIR_TSE_ images. Results imply that there is a correlation between lesion detectability and size in both cases, though. Note. Abbreviations as in Table 7.

The proportions of total TP lesions in both the FLAIR_TSE_ and the corresponding FLAIR_UF_ images are further analyzed in Table 7 and visualized in Figure 11, graded as to size. The sensitivity in terms of lesion detection did not differ significantly between the image variants in large (*p* = 0.68), wide (*p* > 0.99), or narrow (*p* > 0.99) lesion groups. For detecting small lesions, the sensitivity was lower using the FLAIR_UF_ images; however, the difference could not be confirmed statistically (*p* = 0.08).

Presumed causes of FN lesions in both image variants are quantified in Table 8 (large) and Table 9 (small). Causes for lesions not being detected appear to be relatively similar in the FLAIR_UF_ and FLAIR_TSE_ images: (1) lesions were not visible owing to resolution-associated lesion contrast or did not sufficiently stand out from image noise; and (2) lesions were mistaken for cortex or other physiological structures. Table 10 summarizes the presumed causes of FP lesions.

#### 3.2.3. Dependence on Size and Location within FLAIR_UF_

**Figure 12:**
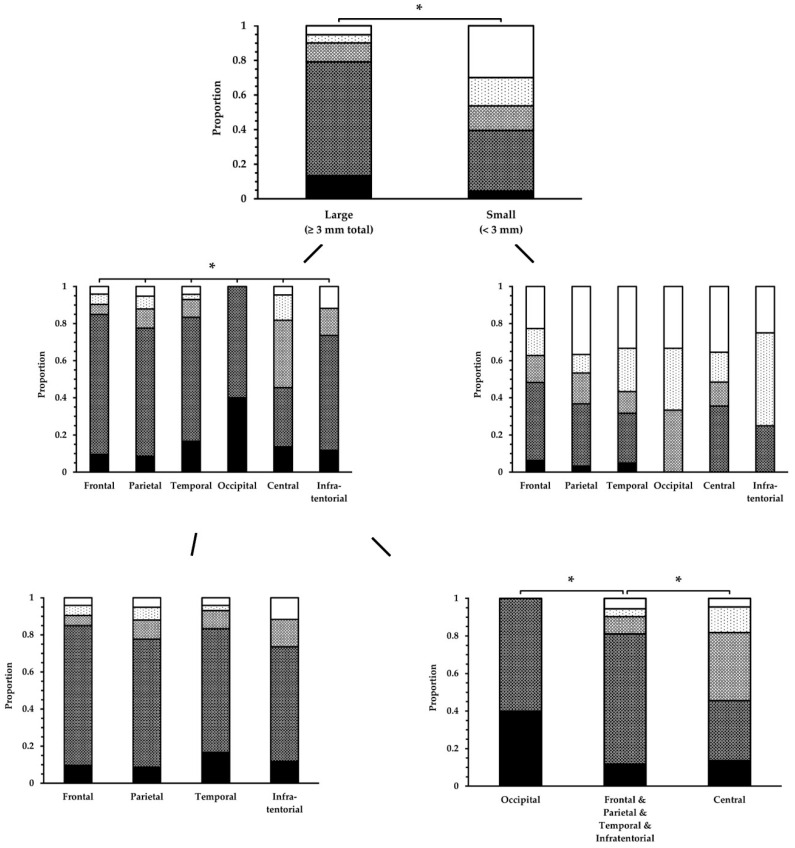
Conspicuity ratings of lesions in the FLAIR_UF_ images, categorized by size and location. Lesion conspicuity was significantly superior for large lesions compared to small lesions. Unless for small lesions, there was a significant difference among some brain regions for large lesions. This could be attributed to occipital lesions (superior) and central lesions (inferior). Note. Black = 1; dark gray = 2; gray = 3; light gray = 4; white = 5. Abbreviations and brain regions as in Table 11. * *p* < 0.05.

Unlike in Section 3.2.1 and Section 3.2.2, the results of ordinal lesion conspicuity ratings of the FLAIR_UF_ images, based on the FLAIR_3Da_ images, are presented in this Section. Comprehensive conspicuity ratings are given in Table 11 and visualized in Figure 12, grouped by lesion size and brain regions. As alluded to in Section 3.2.1, there is a statistically significant difference in terms of lesion conspicuity between large and small FLAIR_UF_ lesions (*p* < 0.001). Testing for conspicuity differences between frontal, parietal, temporal, occipital, central, and infratentorial lesions was significant within the large lesion group (*p* = 0.002), but not among small lesions (*p* = 0.15). Further analysis of large lesions revealed that the differences could be attributed to occipital and central lesions. The conspicuity of large lesions among all the other regions did not differ significantly (*p* = 0.42). Occipital large lesions proved to be more conspicuous (*p* = 0.002) and central large lesions proved to be less conspicuous (*p* = 0.01) compared to all the other large lesions (frontal, parietal, temporal, and infratentorial).

Nevertheless, the results are only valid if they are not biased by other parameters, particularly lesion size variations among the brain regions. Therefore, the average lengthwise lesion diameters are compared in Table 12. The results show that there is no significant or relevant deviation of any group, either for large lesions (*p* = 0.26) or small lesions (*p* = 0.06). Moreover, Table 13 demonstrates the distribution of large lesions between wide and narrow lesions, clustered by location. Also, each group is differentiated by conspicuity ratings. In comparison to all the other locations, there were two brain regions where there were relatively more wide lesions than narrow lesions: occipital and infratentorial. The conspicuity ratings of wide infratentorial lesions appear to be better than the conspicuity ratings of narrow infratentorial lesions; however, that did not hold true for occipital lesions (good ratings without exception).

### 3.3. Image Quality

#### 3.3.1. FLAIR_UF_ Compared with FLAIR_3Da_

**Figure 13:**
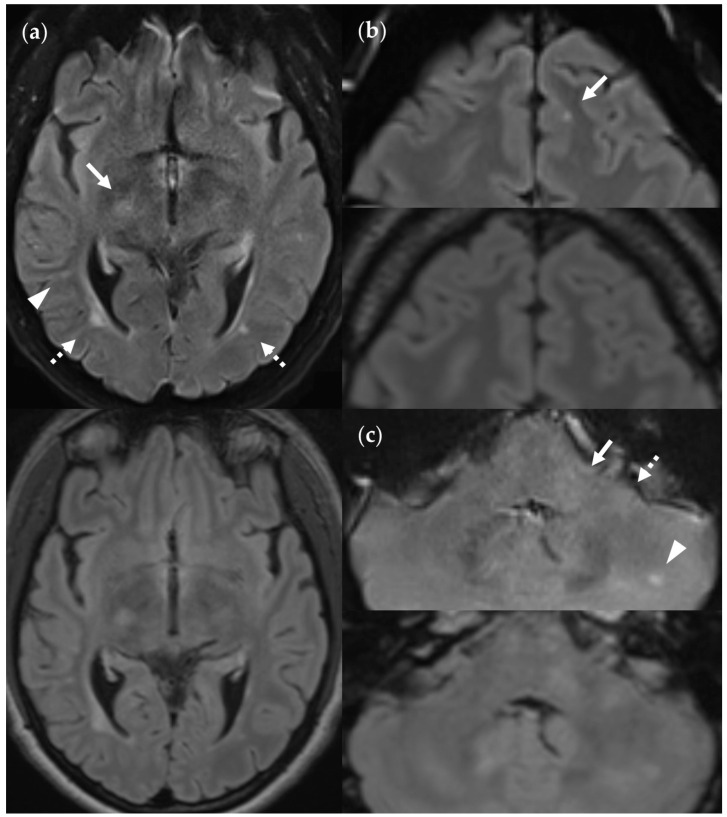
SNR and CNR in FLAIR_UF_ images (top) and FLAIR_3Da_ images (bottom): (**a**) Inflammatory lesions. Continuous arrow: Large lesion in the right mesencephalon (approx. 8 mm × 6 mm). The SNR appeared reduced in the center of the FLAIR_UF_ image, thus decreasing the lesion conspicuity. Dotted arrows: Temporal lesions (right: approx. 5 mm × 4 mm; left: approx. 4 mm × 3 mm). The SNR appeared significantly improved in posterior brain regions in the FLAIR_UF_ images, thus equaling lesion conspicuity between the image variants. Note that in FLAIR_3Da_, the left lesion was better visible in the adjacent image slice (not depicted). Arrowhead: Partially imaged, right temporal lesion (approx. 3 mm × 1 mm in the slice image depicted). Comparison of adjacent slice images showed equal lesion conspicuity. (**b**) Arrow: Left frontal lesion (approx. 2 mm × 1 mm). Excellent lesion conspicuity in the FLAIR_UF_ image due to very good SNR and CNR. (**c**) Infratentorial lesions. Continuous arrow: Large lesion (approx. 9 mm × 6 mm) that was not visible in the FLAIR_UF_ image owing to low SNR and CNR. Dotted arrow: Large lesion (approx. 8 mm × 6 mm) that was less visible in the FLAIR_UF_ image due to reduced SNR and CNR. Arrowhead: Large lesion (approx. 3 mm × 2 mm) that was better visible in the FLAIR_UF_ image. Note that the SNR improves toward the outer regions of the FLAIR_UF_ image.

SNR and CNR in the FLAIR_UF_ images were rated significantly inferior compared to the FLAIR_3Da_ images (*p* < 0.001 each). Rating results are given in Table 14. They represent the overall judgments of the slice series. In fact, there were unequal SNR and CNR levels among the slices, and there was also a positional dependence within one image slice. This is further examined in Section 3.3.3. For image examples, see Figure 13.

**Figure 14:**
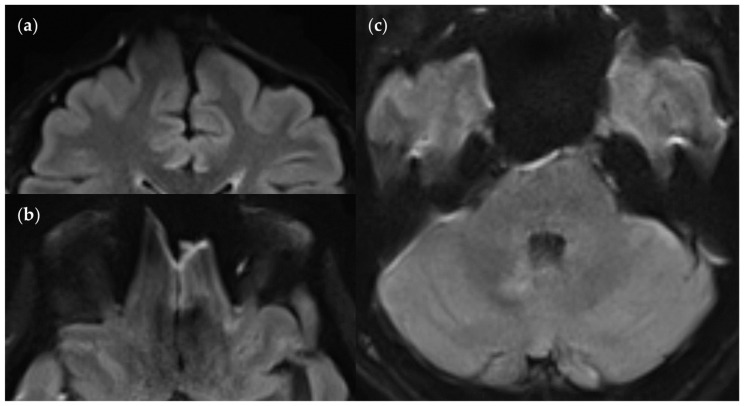
Spatial distortion artifacts in the FLAIR_UF_ images: (**a**) Frontal distortion artifact limiting the diagnostic information in that region. (**b**) Frontobasal distortion artifacts resulting in limited diagnostic information from that region. (**c**) Temporopolar distortion artifacts limiting the diagnostic information in the vicinity. In contrast, the cerebellar and pontine distortion artifacts do not limit diagnostic information.

**Figure 15:**
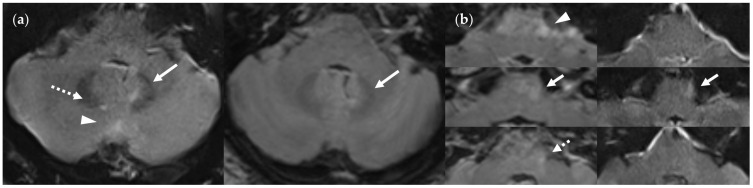
Infratentorial pulsatile flow artifacts that severely limit diagnostic information in the FLAIR_UF_ images and the FLAIR_3Da_ images: (**a**) FLAIR_UF_ (left) and FLAIR_3Da_ as a reference (right), cerebellum: The continuous arrows depict an inflammatory true-positive lesion (approx. 4 mm × 3 mm). The dotted arrow indicates a pulsation artifact that is prone to being confused with a lesion. It was counted as a small, false-positive lesion (approx. 2 mm × 1 mm). The arrowhead points to a pulsation artifact that is not likely to be confused with a lesion due to its typical location adjacent to the occipital sinus. (**b**) FLAIR_3Da_ (left) and FLAIR_UF_ as a reference (right), at the level of the pons: Top images: Typical hyperintense artifact band in the FLAIR_3Da_ image; the arrowhead points to an intensely hyperintense spot within the artifact region that is part of the grainy texture of the artifact. Possible lesions within the artifact region would have been masked completely. Middle images: the continuous arrows show a large lesion (approx. 8 mm × 3 mm) that was misinterpreted as part of the pulsation artifact in the FLAIR_3Da_ image. Bottom images: The dotted arrow denotes a small, false-positive FLAIR_3Da_ lesion (approx. 2 mm × 2 mm) that turned out to be part of the pulsation artifact. Note. Corresponding slices could not be positioned exactly identically owing to different slice thicknesses including slice gaps and non-parallel slice inclinations.

**Figure 16:**
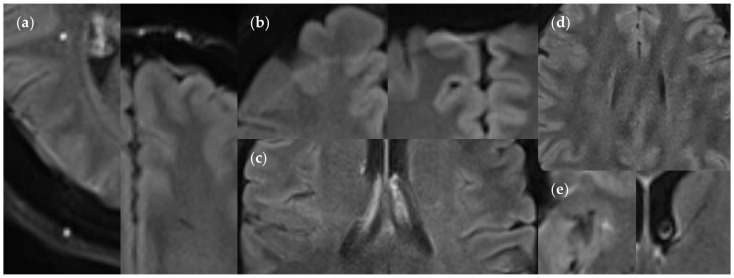
Minor artifacts in the FLAIR_UF_ images: (**a**) Supratentorial pulsation artifacts, caused by extracerebral blood vessels: hyperintense (left image) or hypointense (right image). Hyperintense artifacts can usually be distinguished easily from a lesion, owing to its well-defined, sharply demarcated margin in relation to its size. Hyperintense and hypointense artifacts can usually be related to distant blood vessels, shifted along the phase encoding direction at fixed intervals corresponding to k-space sampling patterns (a quarter of the field of view for the protocol used in our study). Hyperintense artifacts are often located directly adjacent to hypointense artifacts in neighboring image slices. (**b**) Rare chemical shift artifacts due to incomplete fat suppression, in the shape of hyperintense (left image) or hypointense (right image) frontal streaks. (**c**) Rare residual aliasing, in the shape of a subtle hyperintense right central streak. (**d**) Rare spike artifacts, appearing in a herringbone pattern. (**e**) Supratentorial pulsation artifacts, caused by parenchymal blood vessels (singular findings): hyperintense (small FP lesion), insular cortex (left image), and hypointense, anterior limb of internal capsule (right image). They could not be distinguished using the FLAIR_3D_ sequence, however, were clearly correlated with contrast-enhanced images. Also, note the ventricular cerebrospinal fluid pulsation artifact in the right image.

Table 15 lists all artifacts that could be identified in each sequence variant and shows detailed results. Image series were rated positive for artifacts in cases where the artifact could be detected in at least one image slice. First of all, spatial distortions occurred in each of the FLAIR_UF_ sequences, and did not appear in any of the FLAIR_3D_ sequences, representing the main difference. Regions of distortions were as follows: frontal, frontobasal, temporopolar, and infratentorial. As an example, see the images of spatial distortions (Figure 14) as well as the image of a temporopolar distortion-related FN lesion in Figure 7d. Furthermore, we observed infratentorial pulsatile flow artifacts in all sequences, in both the FLAIR_UF_ images and the FLAIR_3Da_ images. Pulsation artifacts (infratentorial and supratentorial) in the FLAIR_UF_ images typically appeared as hyperintense or hypointense dots, spots, or streaks. Pulsation artifacts in the FLAIR_3Da_ images, however, typically appeared as grainy, hyperintense artifact bands positioned at the level of the pons. Limitation of diagnostic information was assumed if there was a tendency or possibility of artifacts mimicking or masking lesions. The former mainly applied to the FLAIR_UF_ images, and the latter mainly pertained to the FLAIR_3Da_ images. Infratentorial pulsation artifacts and related limitations of diagnostic information are demonstrated in Figure 15. Aside from that, we found other infrequent minor artifacts that almost do not affect diagnostic information and occur only in the FLAIR_UF_ images: supratentorial pulsation artifacts, frontal chemical shift artifacts due to incomplete fat suppression, central residual aliasing, and spike artifacts. Plus, there were some motion artifacts in one FLAIR_3Da_ image series that could not be seen in the corresponding FLAIR_UF_ images. Image examples of those minor artifacts are presented in Figure 16.

Finally, it must be pointed out that all analyses were confined to the brain parenchyma. The subarachnoid space, the dura mater, the cranium, and the scalp, along with other parts of the head such as the nasal cavity and the paranasal sinuses, the internal and external ear, the oral cavity, the throat, the eyes, the orbits, the cranial nerves, muscles, adipose tissue, and other osseous or chondral structures, are insufficiently delineated in the FLAIR_UF_ images, as can be seen in the image examples.

#### 3.3.2. FLAIR_UF_ Compared with FLAIR_TSE_

SNR and CNR were rated relatively similar in the FLAIR_UF_ and FLAIR_TSE_ images (see Table 16). However, the low number of paired slice series only serves to provide a rough orientation. Results are overall ratings of the slice series, and positional dependences of SNR and CNR (as demonstrated in Section 3.3.3) seem to be similar in the FLAIR_UF_ and FLAIR_TSE_ images.

**Figure 17:**
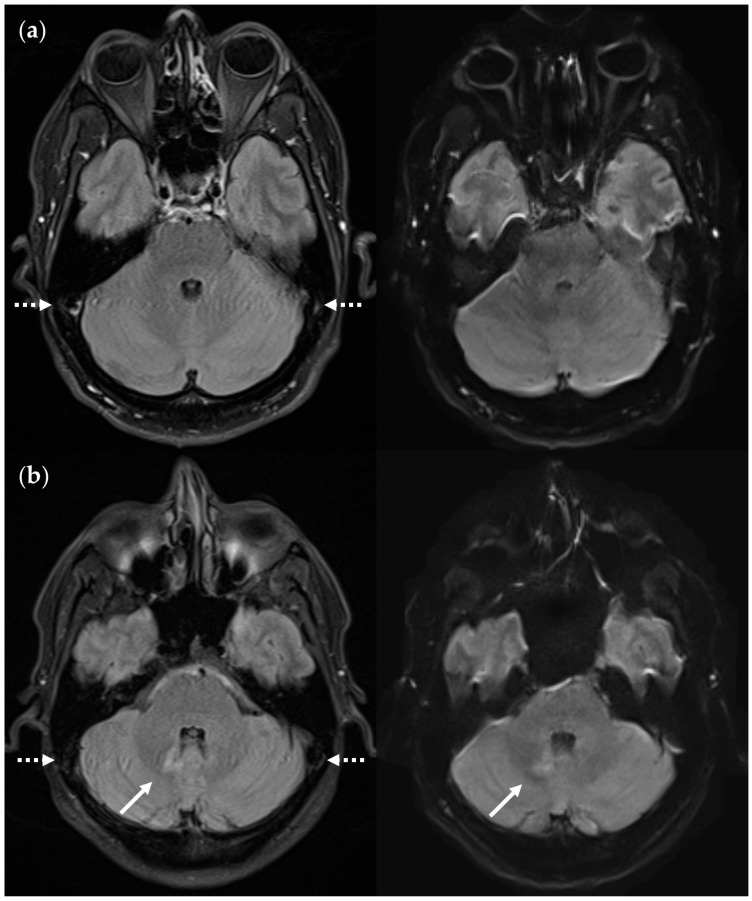
Infratentorial pulsatile flow artifacts in FLAIR_TSE_ (left), contrasted with corresponding FLAIR_UF_ slices (right). The artifact regions are marked with dotted arrows. They appear as irregular, streaky bands traversing the cerebellum from the right sigmoid sinus to the left sigmoid sinus: (**a**) Artifact-induced hyper- and hypointense dots across the cerebellum; the FLAIR_UF_ image confirms that there is no actual lesion in this area. (**b**) Artifact-induced hyper- and hypointense streaks across the cerebellum; the FLAIR_UF_ image confirms that there is one actual lesion (continuous arrow) in this area. Note. Corresponding slices could not be positioned exactly identically owing to different slice intervals and non-parallel slice inclinations.

Artifacts in the FLAIR_UF_ and FLAIR_TSE_ images are contrasted in Table 17. Again, the small amount of data provides only a rough impression. FLAIR_UF_ results are specified in greater detail in Section 3.3.1. Relevant artifacts in the FLAIR_TSE_ images that have the potential to significantly limit diagnostic information are infratentorial pulsatile flow artifacts, either by masking or mimicking lesions. Typically, they appeared as a streaky, irregular (hyperintense and hypointense) band across the middle of the cerebellum, between the right and left sigmoid sinuses (see Figure 17).

#### 3.3.3. Positional Dependence of SNR and CNR in FLAIR_UF_

**Figure 18:**
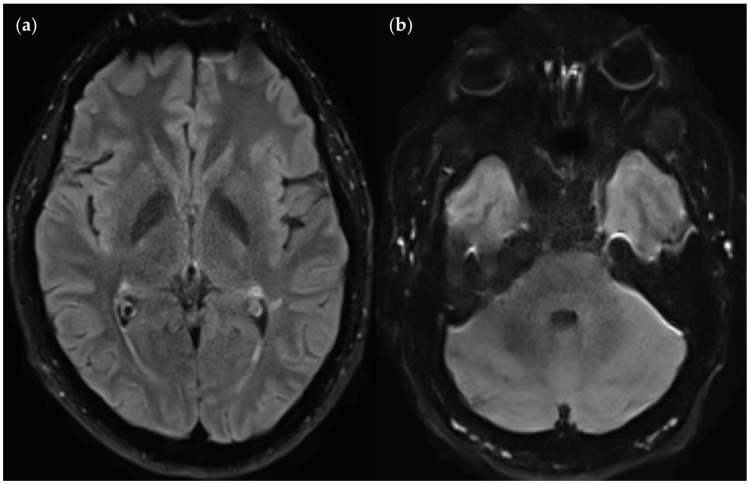
Positional dependence of SNR and CNR within the FLAIR_UF_ images: (**a**) Image slice at the level of the basal nuclei. The SNR deteriorates toward the center of the image, whereas the CNR remains relatively good. Note that both the SNR and CNR are excellent in the marginal regions of the cerebral cortex, e.g., in the occipital lobe. (**b**) Image slice at the level of the pons and the cerebellum. The SNR is substandard around the pons and improves exceedingly toward the posterior lobe of the cerebellum to an excellent level. The CNR, however, seems generally slightly substandard in this area compared to other brain regions, even in the most posterior regions. This may be associated with the characteristic, fine folium-sulcus texture of the cerebellum (e.g., vermis or posterior lobe), which cannot be distinguished clearly.

In the FLAIR_UF_ images, the vicinity of each TP_GS_ lesion was rated for relative SNR and CNR levels (substandard or standard/superior; with reference to the average FLAIR_UF_ image quality). Table 18 and Table 19 present the results grouped by brain regions. Within a region, lesion-rich areas are given more weight than lesion-poor areas. Compared to all the other brain regions, the SNR appeared significantly reduced in central and infratentorial brain regions (*p* < 0.001), and the CNR appeared significantly lower in infratentorial brain regions (*p* < 0.001). What is not evident from the data and is a distinctive characteristic of the infratentorial region is the highly uneven distribution of SNR levels across this area: from highly substandard (in central areas such as the pons) to highly above average (in peripheral areas such as the posterior lobe of the cerebellum). The CNR, however, appeared to be reduced all over the infratentorial areas; particularly, the fine folium-sulcus texture of the cerebellum could not be distinguished sufficiently. Image examples are given in Figure 18.

## 4. Discussion

### 4.1. Main Findings of This Study

This study investigated an ultrafast axial DL-enhanced EPI-FLAIR (FLAIR_UF_) MRI sequence in RRMS patients. The FLAIR_UF_ sequence reduced the acquisition time to almost a sixth of the time required for conventional three-dimensional FLAIR (FLAIR_3D_) acquisitions and nearly a third of the time required for axial standard TSE-FLAIR (FLAIR_TSE_) acquisitions. Based on specific parameters, we investigated lesion detection and image quality in the three sequences. With regard to the detection of inflammatory brain lesions, we did not observe any significant differences between the FLAIR_UF_ images and the FLAIR_3Da_ images in either sensitivity for wide large lesions (≥3 mm × ≥2 mm) or PPV for all large lesions (≥3 mm total) despite the high lesion numbers. The same applies to the sensitivity of large lesion detection (≥3 mm total) in the FLAIR_UF_ images compared to the FLAIR_TSE_ images. Lesion conspicuity did not differ significantly between brain regions in the FLAIR_UF_ images, except for occipital large lesions (superior results) and central large lesions (inferior results). SNR and CNR were significantly inferior using FLAIR_UF_ compared to FLAIR_3D_, which particularly applied to specific areas of the image. In contrast, SNR and CNR in FLAIR_UF_ appeared to be closely comparable to FLAIR_TSE_. Unlike FLAIR_TSE_ and FLAIR_3D_, the image quality in FLAIR_UF_ was limited by distortion artifacts in typical locations. To our knowledge, this study represents the first in-depth quantitative and qualitative evaluation of inflammatory lesions using a DL-enhanced EPI-FLAIR sequence.

### 4.2. Significance of MRI and Ultrafast MRI

In MS patients, the severity of the clinical course is influenced by the start and choice of disease-modifying therapy (DMT). Notably, early treatment start or treatment escalation delays disease progress of both clinically definite multiple sclerosis (CDMS) and clinically isolated syndrome (CIS) in particular [67]. To decide on adequate therapeutic strategies depending on disease activity, MRI plays a major role [10,67,68]. McDonald diagnostic criteria are based on number, location, and size of lesions as well as temporal changes in lesions [6,7,8,9]. A lesion size of over 3 mm is deemed to be appropriate to account for the diagnostic criteria [14]. In order to adapt DMT without delay, specific monitoring intervals are recommended [10]. Notably, CIS patients benefit from very tight follow-up MRI examinations, particularly during the first year [10,69]. Nonetheless, overall MRI scan capacity is far from enough to satisfy demand in numerous parts of the world, and waiting times usually exceed the maximum recommended time, thus worsening medical outcomes and increasing the economic costs [70,71,72,73,74,75]. Against this background, utilizing DL-enhanced EPI-FLAIR sequences may be a promising approach to ameliorate treatment quality while at the same time meeting diagnostic requirements. It might help facilitate low-threshold access to MRI examinations at an earlier stage and at shorter intervals before MS has been diagnosed. Whether FLAIR_UF_ or related techniques could actually be suitable for monitoring the early stages of the disease still requires further investigation.

### 4.3. Limitations of the FLAIR_UF_ Images

Despite its remarkable capabilities, the FLAIR_UF_ images still have three primary limitations. First, lesion size is a limiting factor for the FLAIR_UF_ images, particularly in comparison with the FLAIR_3Da_ images. Almost every second small lesion (<3 mm) and more than every fifth narrow large lesion (≥3 mm × <2 mm) could not be detected using the FLAIR_UF_ images. Additionally, out of all presumed small lesions (<3 mm), about a quarter detected using the ultrafast sequence variant were false-positives. There may be one main cause of these limitations: the relatively large slice thickness including slice gaps in this context. It should be noted that this parameter even influenced the detection of large lesions (≥3 mm).

Secondly, lesion location may represent another limiting factor in the FLAIR_UF_ images. There are several location-dependent factors: (1) SNR deterioration toward the central regions of the images, and simultaneously, SNR enhancement toward marginal regions such as the occipital lobe (even though it appears similar compared with the FLAIR_TSE_ images); (2) some limits of CNR, which primarily become apparent when depicting regions that require high spatial resolution and low slice thickness, particularly the cerebellum with its fine folium-sulcus texture (even though the overall CNR only seems marginally reduced compared with the FLAIR_TSE_ images); (3) pulsatile flow artifacts, which mostly affect image quality by mimicking infratentorial lesions (even though infratentorial pulsation artifacts appear to limit image quality to a similar extent using FLAIR_3Da_ or FLAIR_TSE_, mostly by masking potential lesions); (4) spatial distortion artifacts (frontal, frontobasal, temporopolar, and infratentorial), which may conceal potential lesions; and (5) relatively large slice thickness including slice gaps, which may hinder the distinction between lesions and physiological brain structures at certain locations, particularly near the cortex.

Thirdly, there is an additional limitation apart from those associated with lesion assessment: most structures outside the parenchyma are insufficiently delineated in the FLAIR_UF_ images, such as osseus structures, air-containing structures, or adipose tissue. Reasons for this may be susceptibility artifacts and fat suppression. However, the optic nerves, for example, are clearly distinguishable, and fat suppression might even be advantageous in terms of assessing optic neuritis. However, this remains speculative and beyond the scope of our study.

### 4.4. Considerations on Ratings for Lesion Conspicuity in FLAIR_UF_

Ratings for lesion conspicuity in FLAIR_UF_ (Section 3.2.3) were intended to compare results among the brain regions within the FLAIR_UF_ images. Since a kind of reference was required (FLAIR_3Da_), the ratings appear to be biased by position-dependent lesion conspicuity in FLAIR_3Da_. However, those potential position-dependent differences did not exist at all in the FLAIR_3Da_ images, and image quality was excellent across all regions. The only exception was infratentorial, where a few lesions were masked by pulsation artifacts (FN lesions); all the other infratentorial lesions were not affected in any way. In order to prevent FLAIR_3Da_-biased (infratentorial) conspicuity ratings in FLAIR_UF_, assessments were confined to TP_3Da_ lesions instead of TP_GS_ lesions.

As stated previously, the conspicuity ratings do not seem to be biased by lesion size variations among the brain regions, with one exception: ratings of infratentorial large lesions might have been biased toward falsely positive results, since the proportion of wide lesions to narrow lesions was greater compared to all the other regions, while at the same time, the conspicuity ratings appeared to be superior for wide lesions. That might be an explanation for why the conspicuity ratings do not reflect the substandard SNR and CNR ratings for this region; this discrepancy did not exist in other locations.

Finally, localization-dependent conspicuity results in FLAIR_UF_ could only be demonstrated for large lesions, not for small lesions. The underlying cause could be that slice thickness and slice gaps are the limiting factors for small lesion conspicuity, which is independent of the position. SNR-related positional dependence appears to have a subordinate role in this context. For instance, large central lesions stood out with a comparatively high proportion of grade 3 ratings (conspicuity better in the FLAIR_3Da_ images but classified as a lesion using only the FLAIR_UF_ images), but for small central lesions, that proportion was relatively low in favor of grade 4 and 5 ratings (not classified as a lesion using FLAIR_UF_). In all the other brain regions, on the contrary, large lesions predominantly exhibited grade 1 and 2 ratings (conspicuity at least equal in the FLAIR_UF_ images), i.e., comparatively small proportions of grade 3 ratings, while for small lesions, that proportion was considerably lower in favor of grade 4 and 5 ratings, comparable to the central small lesion group. However, for occipital and infratentorial small lesions, the counts were insufficient to draw further conclusions regarding the position-dependent conspicuity of small lesions.

### 4.5. Outcomes Correlated with Technical Features

The above-mentioned characteristics of the FLAIR_UF_ images arise from specific sequence properties. To begin with, there is a correlation between slice thickness (slice gap included) and visibility of small lesions: if the lesion size is smaller than the slice thickness, the signal intensity will decrease, and with it the lesion contrast due to the partial volume effect. At some point, the lesion signal will disappear completely if the lesions are too small. Thus, there is a direct correlation between the threshold and slice thickness.

Apart from the partial volume effect, EPI-related CNR loss seems to be sufficiently counterbalanced by MT preparation [62]. Here, MT pulses serve as saturation pulses selectively affecting macromolecular protons and adjacent water molecules to enhance image contrast [76].

In EPI, the SNR is diminished due to substantial signal loss during its long echo train, and lack of refocusing pulses combined with rapid T2* signal decay. Nevertheless, aided by the DL reconstruction, the SNR in FLAIR_UF_ appeared similar compared to FLAIR_TSE_. In both image variants, the SNR (and related lesion conspicuity) decreased similarly toward the center of the image, owing to coil dependency in PI [77,78,79]. Accordingly, there is an inversely proportional relationship between SNR and the geometry (g)-factor [77,78,79]. This factor is affected by coil sensitivity and voxel location, usually exhibiting the greatest values in the center of the image [77,78,79]. Although the DL reconstruction method was designed to compensate for this specific factor, its SNR gains were eventually limited, especially in combination with stringent requirements for slice thickness. In contrast, the excellent SNR in FLAIR_3D_ can be attributed to the fact that in 3D acquisitions, the complete volume is excited with each shot.

In addition, the long readout duration in EPI has another significant impact on image quality (together with the very low pixel bandwidth along the phase-encoding direction in EPI): increased sensitivity to susceptibility artifacts [80]. If a substance is located in an external homogeneous magnetic field, the magnetic field lines will either be dispersed (e.g., bone tissue) or concentrated (e.g., air or metal) around that material, depending on its susceptibility properties [81]. Consequently, the magnetic field is disturbed in areas where susceptibility differences are large (e.g., air-filled bones), leading to an accelerated phase coherence loss and T2* signal decay, on the one hand, and accumulation of phase errors along with positioning errors in the phase encoding direction, on the other [2]. This explains the appearance of both distortion artifacts, particularly arising in the vicinity of air–tissue interfaces in the phase encoding direction, and signal decrease in tissues surrounding the brain parenchyma, caused by differences in magnetic susceptibility. In strong magnetic fields, susceptibility differences further increase, and phase incoherences accumulate to a greater extent over the duration of the echo train [2,82,83]. In contrast, SNR, CNR, spatial resolution, and scan time benefit from a strong magnetic field strength [82,83].

Apart from susceptibility effects, there is another reason for the signal decrease in non-parenchymal tissues in FLAIR_UF_: fat suppression. It is essential for low-segmented EPI in order to avert chemical shift artifacts [2]. Those artifacts derive from spatial signal misregistration owing to frequency differences between protons in fat and water. Without fat suppression, however, the implicitly low pixel bandwidth along the phase-encoding direction in EPI would lead to the accumulation of phase shifts between fat and water, resulting in chemical shift artifacts in the phase-encoding direction [80,84]. With singular exceptions located close to the frontal air–tissue interfaces, we did not observe any chemical shift artifacts caused by incomplete fat suppression in the FLAIR_UF_ images.

In addition, we observed other artifacts caused by phase errors: pulsatile flow artifacts. They usually occur along the phase encoding direction [85]. Accordingly, we were able to relate most artifacts to structures containing flowing blood or liquor in all three image variants (FLAIR_UF_, FLAIR_TSE_, and FLAIR_3Da_), each of them revealing distinctive characteristics. Typically, the artifacts either occurred around blood vessels, particularly around infratentorial venous sinuses (FLAIR_UF_), or they were aligned in positions shifted from blood vessels along the phase encoding direction (FLAIR_UF_, FLAIR_3Da_, and FLAIR_TSE_). In the FLAIR_UF_ and FLAIR_3Da_ images, artifacts were shifted along the anterior–posterior axis, while in the FLAIR_TSE_ images, artifacts were shifted along the left–right axis (compare Table 2). In the FLAIR_UF_ images, replicas were shifted at intervals of a quarter of the field of view (according to the PI acceleration factor of two and the two-segmented k-space). In the FLAIR_3Da_ and FLAIR_TSE_ images, artifacts manifested as grainy or streaky bands. However, in terms of limitations of diagnostic information caused by pulsation artifacts, the FLAIR_UF_ images were not inferior.

Finally, there were three more rare minor artifacts, two of which could be seen in the FLAIR_UF_ images: residual aliasing and spike artifacts. The former is related to PI reconstruction, and the latter is related to the rapid switching of gradients in EPI. The third artifact type, minor motion artifacts caused by subject movement, could not be observed in the FLAIR_UF_ images, but rather in one FLAIR_3Da_ and one FLAIR_TSE_ image series. This is related to the longer acquisition time in FLAIR_3D_ and FLAIR_TSE_. In this regard, EPI is undeniably at an advantage.

### 4.6. Limitations of the Study

There are some limitations of this study that might have affected our results. To begin with, the methodological approach may be associated with some selection bias. Examinations were carried out using one specific scanner, and the patient sample may be biased by common appointment scheduling practices in terms of disease manifestation or severity (medium or low disease activity in an outpatient follow-up setting). Although no patient declined to participate in the study, there were a couple of patients who could not be included from the outset due to the associated additional expenditure of time. That probably led to further sampling bias (e.g., age, sex, clinical condition). In particular, the sex ratio in our study was atypical (compare Section 1 and Section 2.1). Aside from selection bias, there were more factors limiting the external validity, such as the single-center study design using only one single 3 T MRI scanner from one specific manufacturer. In consideration of our methodological objective to analyze lesion detection and image quality in general, it must be pointed out that the comparisons performed could only be based on limited parameters.

Moreover, there might have been some observer bias inherent to this study, despite all efforts and critical appraisal. In this regard, implementing image blinding would not have had a positive effect due to the significantly different and easily distinguishable nature of the images, and the study was designed for subjective side-by-side comparisons. Generally, radiological readers’ assessments may also depend on their level of experience with the FLAIR_UF_ image characteristics.

Even though the number of participants was modest, the lesion counts were substantial, providing high statistical power. Nevertheless, there is an important limitation from a statistical point of view: statistically, several lesions within one subject are not independent observations, because they are bound to both the same individual and time of scan. Accordingly, lesion conspicuity could possibly have been influenced by parameters that might have varied among the individuals or scans, which may have induced statistical bias. Also, non-significant test results are indications, but not proof, of equality. So, in large part, the results of the study are indicative, not conclusive. However, variances among the image data appeared to play a minor role, at most, and non-significant results are more meaningful when based on a large sample size. Methodologically, it would have been precise either to rate all lesions from one image series as a whole or to randomly select and analyze only one lesion from each image series after recording all existing lesions. However, the former procedure would have provided highly inaccurate outcomes, and the latter procedure would have provided relatively meaningless test results (if statistically non-significant), owing to the sample size. In this study, the small FLAIR_TSE_ sample size only provides indicative findings: results were either statistically tested, accepting the possibility of an increased type II error, or statistical testing was omitted.

While the acquisition sequence used in this study would allow for reducing the slice thickness to 3 mm without compromising scan coverage and total scan time, it remains unclear to what extent DL processing may compensate for the corresponding 25% reduction in SNR. Assessing further optimizations of the acquisition protocol may represent a rewarding task for future studies. Also, further prospective studies and confirmatory trials with larger numbers of participants and more diverse cohorts, various scanners, and modified reconstruction and acquisition methods need to be conducted. Further radiological reading should possibly include separate image assessments instead of side-by-side comparisons. Plus, other studies covering MS patients might consider additional contrasts (e.g., contrast-enhanced T1w sequences), orientations (sagittal, coronal), or regions (such as the spinal cord or the optic nerves).

### 4.7. Future Perspectives

All in all, our results suggest that FLAIR_UF_ may be an appropriate approach for assessing cerebral inflammatory lesions. Diagnostic performance did not prove inferior compared to conventional FLAIR_3D_ in terms of MS lesion detection (≥3 mm × ≥2 mm) in just about a sixth of the time. This demonstrates the tremendous progress made in the field of EPI. In light of extensive waiting times for MRI examinations and patient-dependent constraints to carry them out, for example, claustrophobia or inability to remain motionless (i.e., children or disease- and age-related obstacles), ultrafast imaging seems an essential tool. However, there are still some challenges to overcome in refining this technique while aiming to combine high resolution with high SNR at high field strengths along with low artifacts.

## 5. Conclusions

In conclusion, this study indicates that ultrafast DL-enhanced EPI-FLAIR might be an appropriate method for the assessment of cerebral inflammatory lesions (≥3 mm) in MS. Apart from relatively minor limitations regarding lesion size and lesion location, it did not prove inferior to conventional FLAIR methods in the main, while being conducted in a fraction of time. In consideration of both MRI waiting times and patients who cannot remain motionless in the scanner for long, EPI might be an expedient alternative to conventional imaging. However, further studies are required to confirm our findings.

## Figures and Tables

**Table 1:** Patient characteristics.

Characteristics	Values
Number of patients	17
Mean age ± standard deviation	33 ± 10 years
Median age (range)	29 (21–60) years
Distribution between sexes	71% male (*n* = 12), 29% female (*n* = 5)

Note. One patient underwent a second follow-up MRI examination six months later during the investigation period and is included twice. Years and percentages are rounded to the whole numbers.

**Table 2:** MRI acquisition parameters.

Parameter	FLAIR_UF_	FLAIR_TSE_	FLAIR_3D_
Orientation	Axial	Axial	-
Sequence type	Multi-shot EPI	TSE	SPACE
TR (ms)	8000	8800	5000
TE (ms)	88	87	386
TI (ms)	2372	2480	1800
Flip angle (°)	180	150	120 (VFA)
Voxel size (mm)	0.9 × 0.9 × 4	0.7 × 0.7 × 4	0.5 × 0.5 × 0.9 ^1^
Gap between slices (mm)	0.8	0	-
Phase encoding direction	P → A	R → L	A → P
Acceleration mode	DL-based	GRAPPA	GRAPPA
Acceleration factor	2	2	2
In-plane FOV (read × phase; mm)	230 × 230	230 × 187	256 × 256 ^1^
Number of slices	32	36	192
Time of acquisition (min:s)	0:51	2:22	4:57

Note. FLAIR = fluid-attenuated inversion recovery; FLAIR_UF_ = ultrafast FLAIR sequence; FLAIR_TSE_ = turbo/fast spin-echo FLAIR sequence; FLAIR_3D_ = three-dimensional FLAIR sequence; TR = repetition time; TE = echo time; TI = inversion time; FOV = field-of-view; EPI = echo-planar-imaging; TSE = turbo/fast spin echo; SPACE = sampling perfection with application-optimized contrasts using different flip angle evolution; VFA = variable flip angles; A = anterior; P = posterior; R = right; L = left; DL = deep learning; GRAPPA = generalized autocalibrating partial parallel acquisition. ^1^ Values refer to the sagittal orientation. Slice thickness of the axially reconstructed images used for side-by-side comparisons (FLAIR_3Da_) = 3 mm.

**Table 3:** Lesion detection in FLAIR_UF_ images and FLAIR_3Da_ images.

		Total				Sensitivity		PPV
Lesion Size	S	TP	FN	TP_GS_	FP	95% CI [LL, UL]	*p*	95% CI [LL, UL]
		*n*	*n*	*n*	*n*	*%*		*%*
Large (≥3 mm)	UF	251	37	288	11	87.2 [82.7, 90.8]	<0.001	95.8 [92.6, 97.9]
	3Da	274	14		11	95.1 [92.0, 97.3]		96.1 [93.2, 98.1]
Wide (×≥2 mm)	UF	160	11	171	6	93.6 [88.8, 96.7]	0.50	96.4 [92.3, 98.7]
	3Da	163	8		8	95.3 [91.0, 98.0]		95.3 [91.0, 98.0]
Narrow (×<2 mm)	UF	91	26	117	5	77.8 [69.2, 84.9]	<0.001	94.8 [88.3, 98.3]
	3Da	111	6		3	94.9 [89.2, 98.1]		97.4 [92.5, 99.5]
Small (<3 mm)	UF	148	132	280	33	52.9 [46.8, 58.8]	<0.001	81.8 [75.4, 87.1]
	3Da	268	12		15	95.7 [92.6, 97.8]		94.7 [91.4, 97.0]

Note. S = sequence; UF = FLAIR_UF_; 3Da = FLAIR_3Da_; PPV = positive predictive value; CI = confidence interval (Clopper–Pearson); LL = lower limit; UL = upper limit; further abbreviations as in Figure 2, Figure 3 and Figure 4. To compare sensitivity results (or, more precisely, to compare the FN_UF_ and TP_3Da_ lesion counts with the FN_3Da_ and TP_UF_ lesion counts) McNemar’s test was performed, based on paired data shown in Figure 5. To compare PPV results, no statistical test was performed, since data were neither completely paired nor completely independent samples.

**Table 4:** Presumed causes of FN large lesions (≥3 mm total) in FLAIR_UF_ and FLAIR_3Da_ images.

		FLAIR_UF_			FLAIR_3Da_		
Presumed Type of Cause		FN_UF_ Total	FN_UF_ and TP_3Da_	FN_UF_ and FN_3Da_		FN_3Da_ and TP_UF_	FN_3Da_ Total
		*n*	*n*	*n*	*n*	*n*	*n*
Not detectable	SR/CNR/SNR ^2^	28	22	6	0	1	1
Mistaken for natural structure ^1^		7	4	3	3	0	3
Mistaken for/masked by pulsation artifact		0	0	0	7 ^3^	3 ^3^	10 ^3^
Masked by distortion artifact		2	1	1	0	0	0
Total		37	27	10		4	14

Note. SR = spatial resolution, esp. slice thickness; CNR = contrast-to-noise ratio; SNR = signal-to-noise ratio; further abbreviations as in Figure 2 and Figure 4. ^1^ Cortex, in particular. ^2^ Causes of SR, CNR, and SNR could not strictly be distinguished. It is likely that there is a combination of those causes in most cases. ^3^ All lesions were located in an infratentorial position.

**Table 5:** Presumed causes of FN small lesions (<3 mm) in FLAIR_UF_ and FLAIR_3Da_ images.

		FLAIR_UF_			FLAIR_3Da_		
Presumed Type of Cause		FN_UF_ Total	FN_UF_ and TP_3Da_	FN_UF_ and FN_3Da_		FN_3Da_ and TP_UF_	FN_3Da_ Total
		*n*	*n*	*n*	*n*	*n*	*n*
Not detectable	SR/CNR/SNR ^2^	110	105	5	0	3	3
Mistaken for natural structure ^1^		21	19	2	3	1	4
Mistaken for/masked by pulsation artifact		0	0	0	5 ^3^	0	5 ^3^
Masked by distortion artifact		1	0	1	0	0	0
Total		132	124	8		4	12

Note. Abbreviations as in Table 4. ^1^ Cortex, in particular. ^2^ Causes of SR, CNR, and SNR could not strictly be distinguished. It is likely that there is a combination of those causes in most cases. ^3^ Three were located in an infratentorial position; the rest were located in a supratentorial position.

**Table 6:** Presumed causes of FP lesions in FLAIR_UF_ and FLAIR_3Da_ images.

		FLAIR_UF_			FLAIR_3Da_		
Presumed Causal Phenomenon		FP_UF_ Large (≥3 mm)	FP_UF_ Small (<3 mm)	FP_UF_ Total	FP_3Da_ Large (≥3 mm)	FP_3Da_ Small (<3 mm)	FP_3Da_ Total
		*n*	*n*	*n*	*n*	*n*	*n*
Partially imaged natural structure ^1^	SR ^2^	9	24	33	6	7	13
Partially imaged nearby large lesion		0	2	2	0	1	1
Pulsation artifact		2 ^3^	7 ^3^	9 ^3^	5 ^4^	7 ^5^	12 ^5^
Total		11	33	44	11	15	26

Note. SR = spatial resolution, esp. slice thickness; FP_UF_ = false-positive lesions using the FLAIR_UF_ images; FP_3Da_ = false-positive lesions using the FLAIR_3Da_ images. ^1^ Cortex, in particular. ^2^ CNR and SNR are also partly involved. ^3^ One was located in a supratentorial position; the rest were located in an infratentorial position. ^4^ All lesions were located in an infratentorial position. ^5^ Three were located in a supratentorial position; the rest were located in an infratentorial position.

**Table 7:** Lesion detectability in FLAIR_TSE_ images and FLAIR_UF_ counterparts.

		Total			Sensitivity	
Lesion Size	S	tseTP	tseFN	tseTP_GS_	95% CI [LL, UL]	*p*
		*n*	*n*	*n*	*%*	
Large (≥3 mm)	UF	48	5	53	90.6 [79.3, 96.9]	0.68
	TSE	48	5		90.6 [79.3, 96.9]	
Wide (×≥2 mm)	UF	33	2	35	94.3 [80.8, 99.3]	>0.99
	TSE	34	1		97.1 [85.1, 99.9]	
Narrow (×<2 mm)	UF	15	3	18	83.3 [58.6, 96.4]	>0.99
	TSE	14	4		77.8 [52.4, 93.6]	
Small (<3 mm)	UF	19	15	34	55.9 [37.9, 72.8]	0.08
	TSE	27	7		79.4 [62.1, 91.3]	

Note. TSE = FLAIR_TSE_; further abbreviations as in Table 3 and Figure 8. To compare sensitivity results, McNemar’s test was performed, based on paired data shown in Figure 9. Unlike in the previous Section, the corresponding FP values (see Table 10) were insufficient to ensure reliable PPV analysis results; hence, it was omitted here.

**Table 8:** Presumed causes of FN large lesions (≥3 mm total) in FLAIR_TSE_ and corresponding FLAIR_TSE_ images.

		FLAIR_UF_			FLAIR_TSE_		
Presumed Type of Cause		tseFN_UF_ Total	tseFN_UF_ and TP_TSE_	tseFN_UF_ and FN_TSE_		FN_TSE_ and tseTP_UF_	FN_TSE_ Total
		*n*	*n*	*n*	*n*	*n*	*n*
Not detectable	SR/CNR/SNR ^2^	3	3	0	0	3	3
Mistaken for natural structure ^1^		2	0	2	2	0	2
Total		5	3	2		3	5

Note. Abbreviations as in Table 4 and Figure 8. ^1^ Cortex, in particular. ^2^ Causes of SR, CNR, and SNR could not strictly be distinguished. It is likely that there is a combination of those causes in most cases.

**Table 9:** Presumed causes of FN small lesions (<3 mm) in FLAIR_TSE_ and corresponding FLAIR_TSE_ images.

		FLAIR_UF_			FLAIR_TSE_		
Presumed Type of Cause		tseFN_UF_ Total	tseFN_UF_ and TP_TSE_	tseFN_UF_ and FN_TSE_		FN_TSE_ and tseTP_UF_	FN_TSE_ Total
		*n*	*n*	*n*	*n*	*n*	*n*
Not detectable	SR/CNR/SNR ^2^	13	10	3	3	3	6
Mistaken for natural structure ^1^		2	2	0	0	1	1
Total		15	12	3		4	7

Note. Abbreviations as in Table 4 and Figure 8. ^1^ Cortex, in particular. ^2^ Causes of SR, CNR, and SNR could not strictly be distinguished. It is likely that there is a combination of those causes in most cases.

**Table 10:** Presumed causes of FP lesions in FLAIR_UF_ and FLAIR_TSE_ images.

		FLAIR_UF_			FLAIR_TSE_		
Presumed Causal Phenomenon		tseFP_UF_ Large (≥3 mm)	tseFP_UF_ Small (<3 mm)	tseFP_UF_ Total	FP_TSE_ Large (≥3 mm)	FP_TSE_ Small (<3 mm)	FP_TSE_ Total
		*n*	*n*	*n*	*n*	*n*	*n*
Partially imaged natural structure ^1^	SR ^2^	1	1	2	1	3	4
Partially imaged nearby large lesion		0	1	1	0	1	1
Total		1	2	3	1	4	5

Note. SR = spatial resolution, esp. slice thickness; FP_TSE_ = false-positive lesions using the FLAIR_TSE_ images; tseFP_UF_ = corresponding subset of FP_UF_ recorded with FLAIR_TSE_. ^1^ Cortex, in particular. ^2^ CNR and SNR are also partly involved.

**Table 11:** Conspicuity ratings of lesions in the FLAIR_UF_ images, categorized by size and location.

		Conspicuity						
Size	Location	1	2	3	4	5	TP_3Da_	*p*
		*n*	*n*	*n*	*n*	*n*	*n*	
Large (≥3 mm total)	All	37	180	30	13	14	274	<0.001 ^3^
Small (<3 mm)	All	12	94	38	44	80	268	
Large (≥3 mm total)	Frontal	7	55	4	4	3	73	0.002 ^4^
	Parietal	5	40	6	4	3	58	
	Temporal	12	48	7	2	3	72	
	Occipital	6	9	0	0	0	15	
	Central ^1^	3	7	8	3	1	22	
	Infratentorial ^2^	4	21	5	0	4	34	
Small (<3 mm)	Frontal	7	46	16	16	25	110	0.15 ^4^
	Parietal	2	20	10	6	22	60	
	Temporal	3	16	7	14	20	60	
	Occipital	0	0	1	1	1	3	
	Central ^1^	0	11	4	5	11	31	
	Infratentorial ^2^	0	1	0	2	1	4	
Large (≥3 mm total)	Frontal	7	55	4	4	3	73	0.42 ^4^
	Parietal	5	40	6	4	3	58	
	Temporal	12	48	7	2	3	72	
	Infratentorial ^2^	4	21	5	0	4	34	
Small (≥3 mm total)	Occipital	6	9	0	0	0	15	
								0.002 ^3^
	Frontal and Parietal and Temporal and Infratentorial ^2^	28	164	22	10	13	237	
								0.01 ^3^
	Central ^1^	3	7	8	3	1	22	

Note. TP_3Da_ = true-positive lesion counts using FLAIR_3Da_. The counts of lesions are given. The conspicuity was rated on an ordinal five-point Likert scale, based on the FLAIR_3Da_ images: 1 = better/larger in the FLAIR_UF_ images; 2 = equal; 3 = better in the FLAIR_3Da_ images but classified as a lesion using only the FLAIR_UF_ images; 4 = better in the FLAIR_3Da_ images and classified as no lesion using only the FLAIR_UF_ images; 5 = FLAIR_3Da_ lesion that is not visible at all in the FLAIR_UF_ images. ^1^ Insular lobe, corpus callosum, basal nuclei, and diencephalon. ^2^ Brainstem and cerebellum. ^3^ Wilcoxon rank-sum test. ^4^ Kruskal–Wallis test.

**Table 12:** Average lesion diameters (lengthwise), grouped by size category and location.

Location	Large (≥3 mm total)					Small (<3 mm)				
	TP_3Da_	M and 95% CI [LL, UL]	SD	Mdn	*p*	TP_3Da_	M and 95% CI [LL, UL]	SD	Mdn	*p*
	*n*	mm	mm	mm		*n*	mm	mm	mm	
Frontal	73	5.1 [4.6, 5.6]	1.9	4.6	0.26	110	2.1 [2.0, 2.2]	0.5	2.3	0.06
Parietal	58	5.8 [5.0, 6.7]	3.2	4.6		60	2.1 [1.9, 2.2]	0.6	2.1	
Temporal	72	5.3 [4.7, 5.9]	2.5	4.6		60	2.0 [1.8, 2.1]	0.6	2.1	
Occipital	15	5.6 [4.6, 6.6]	1.8	5.4		3	2.8 [2.6, 2.9]	0.1	2.8	
Central	22	5.5 [3.8, 7.2]	3.8	4.5		31	2.2 [2.1, 2.4]	0.5	2.4	
Infratentorial	34	6.5 [5.4, 7.5]	3.0	5.2		4	2.3 [1.9, 2.8]	0.3	2.4	
Total	274	5.5 [5.2, 5.9]	2.7	4.7		268	2.1 [2.0, 2.2]	0.6	2.2	

Note. M = mean; CI = confidence interval; LL = lower limit; UL = upper limit; SD = standard deviation; Mdn = median; further abbreviations and brain regions as in Table 11. Since the assumptions of normal distribution of the data were not met, a Kruskal–Wallis test was performed.

**Table 13:** Conspicuity ratings of large lesions (≥3 mm), grouped by width category and location.

Location	Wide (≥3 mm × ≥2 mm)						Narrow (≥3 mm × <2 mm)						Large (≥3 mm Total)
	Conspicuity					Total	Conspicuity					Total	TP_3Da_
	1	2	3	4	5		1	2	3	4	5		
	*n*	*n*	*n*	*n*	*n*	*n*	*n*	*n*	*n*	*n*	*n*	*n*	*n*
Frontal	7	35	0	0	1	43	0	20	4	4	2	30	73
Parietal	2	28	2	1	0	33	3	12	4	3	3	25	58
Temporal	5	30	4	1	0	40	7	18	3	1	3	32	72
Occipital	4	8	0	0	0	12	2	1	0	0	0	3	15
Central	0	4	5	1	0	10	3	3	3	2	1	12	22
Infratentorial	3	17	3	0	2	25	1	4	2	0	2	9	34
Total	21	122	14	3	3	163	16	58	16	10	11	111	274

Note. The counts of lesions are given. Abbreviations and brain regions as in Table 11.

**Table 14:** SNR and CNR in FLAIR_UF_ and FLAIR_3Da_ images.

Parameter	FLAIR_UF_			FLAIR_3Da_			*p*
	Mdn (IQR)	M ± SD	L/H	Mdn (IQR)	M ± SD	L/H	
SNR	3 (3–3)	3.00 ± 0.00	3/3	1 (1–1)	1.18 ± 0.39	1/2	<0.001
CNR	3 (2–3)	2.53 ± 0.51	2/3	1 (1–1)	1.18 ± 0.39	1/2	<0.001

Note. N = 17. SNR = signal-to-noise ratio; CNR = contrast-to-noise ratio; Mdn = median; IQR = interquartile range; M = mean; SD = standard deviation; L = lowest value; H = highest value. Both parameters were rated on an ordinal five-point Likert scale: 1 = very good; 2 = good; 3 = acceptable; 4 = mediocre/diagnostic; 5 = poor/non-diagnostic. The *p*-values were calculated using the Wilcoxon signed-rank test.

**Table 15:** Artifacts in the FLAIR_UF_ and FLAIR_3Da_ images.

Artifacts	FLAIR_UF_			FLAIR_3Da_			*p*
	Mdn (IQR)	M ± SD	L/H	Mdn (IQR)	M ± SD	L/H	
Distortions							
Frontal	3 (2–3)	2.6 ± 0.6	1/3	0 (0–0)	0.0 ± 0.0	0/0	<0.001
Frontobasal	3 (2–3)	2.7 ± 0.5	2/3	0 (0–0)	0.0 ± 0.0	0/0	<0.001
Temporopolar	3 (2.5–3)	2.8 ± 0.4	2/3	0 (0–0)	0.0 ± 0.0	0/0	<0.001
Infratentorial	1 (1–1)	1.1 ± 0.3	1/2	0 (0–0)	0.0 ± 0.0	0/0	<0.001
Pulsatile flow							
infratentorial	1 (1–4)	2.2 ± 1.6	0/4	3 (3–4)	3.4 ± 0.7	2/4	0.02
supratentorial	1 (0–1)	0.7 ± 0.8	0/3	0 (0–0)	0.0 ± 0.0	0/0	0.004
Chemical shift							
Frontal	0 (0–0)	0.1 ± 0.3	0/1	0 (0–0)	0.0 ± 0.0	0/0	0.16
Residual Aliasing							
Central	0 (0–0)	0.2 ± 0.6	0/2	0 (0–0)	0.0 ± 0.0	0/0	0.10
Spikes ^1^	0 (0–1)	0.4 ± 0.6	0/2	0 (0–0)	0.0 ± 0.0	0/0	0.03
Subject motion	0 (0–0)	0.0 ± 0.0	0/0	0 (0–0)	0.1 ± 0.2	0/1	0.32

Note. N = 17. Mdn = median; IQR = interquartile range; M = mean; SD = standard deviation; L = lowest value; H = highest value. All artifacts were rated on an ordinal five-point Likert scale: 0 = no artifact; 1 = artifact exists, but diagnostic information is not limited; 2 = artifact exists, and diagnostic information is slightly limited in the artifact region; 3 = artifact exists, and diagnostic information is limited in the artifact region; 4 = artifact exists, and diagnostic information is severely limited in the artifact region. The *p*-values were calculated using the Wilcoxon signed-rank test. ^1^ In the case of a positive rating, artifacts only occurred in one single image slice each.

**Table 16:** SNR and CNR in FLAIR_UF_ and FLAIR_TSE_ images.

Parameter	FLAIR_UF_			FLAIR_TSE_		
	Mdn (IQR)	M ± SD	L/H	Mdn (IQR)	M ± SD	L/H
SNR	3 (3–3)	3.00 ± 0.00	3/3	3 (3–3)	3.00 ± 0.00	3/3
CNR	3 (2.5–3)	2.67 ± 0.58	2/3	2 (2–2)	2.00 ± 0.00	2/2

Note. N = 3. SNR = signal-to-noise ratio; CNR = contrast-to-noise ratio; Mdn = median; IQR = interquartile range; M = mean; SD = standard deviation; L = lowest value; H = highest value. Both parameters were rated on an ordinal five-point Likert scale: 1 = very good; 2 = good; 3 = acceptable; 4 = mediocre/diagnostic; 5 = poor/non-diagnostic. Significance testing was omitted due to the low number of cases.

**Table 17:** Artifacts in the FLAIR_UF_ and FLAIR_TSE_ images.

Artifacts	FLAIR_UF_			FLAIR_TSE_		
	Mdn (IQR)	M ± SD	L/H	Mdn (IQR)	M ± SD	L/H
Distortions						
Frontal	3 (3–3)	3.0 ± 0.0	3/3	0 (0–0)	0.0 ± 0.0	0/0
Frontobasal	3 (3–3)	3.0 ± 0.0	3/3	0 (0–0)	0.0 ± 0.0	0/0
Temporopolar	3 (3–3)	3.0 ± 0.0	3/3	0 (0–0)	0.0 ± 0.0	0/0
Infratentorial	1 (1–1)	1.0 ± 0.0	1/1	0 (0–0)	0.0 ± 0.0	0/0
Pulsatile flow						
infratentorial	1 (1–1)	1.0 ± 0.0	1/1	2 (1.5–3)	2.3 ± 1.5	1/4
supratentorial	0 (0–0)	0.0 ± 0.0	0/0	0 (0–0.5)	0.3 ± 0.6	0/1
Chemical shift						
Frontal	0 (0–0)	0.0 ± 0.0	0/0	0 (0–0)	0.0 ± 0.0	0/0
Residual Aliasing						
Central	0 (0–1)	0.7 ± 1.2	0/2	0 (0–0)	0.0 ± 0.0	0/0
Spikes ^1^	0 (0–0.5)	0.3 ± 0.6	0/1	0 (0–0)	0.0 ± 0.0	0/0
Subject motion	0 (0–0)	0.0 ± 0.0	0/0	0 (0–0.5)	0.3 ± 0.6	0/1

Note. N = 3. Mdn = median; IQR = interquartile range; M = mean; SD = standard deviation; L = lowest value; H = highest value. All artifacts were rated on an ordinal five-point Likert scale: 0 = no artifact; 1 = artifact exists, but diagnostic information is not limited; 2 = artifact exists, and diagnostic information is slightly limited in the artifact region; 3 = artifact exists, and diagnostic information is limited in the artifact region; 4 = artifact exists, and diagnostic information is severely limited in the artifact region. Significance testing was omitted due to the low number of cases. ^1^ In the case of a positive rating, artifacts only occurred in one single image slice each.

**Table 18:** FLAIR_UF_: SNR assessment in the vicinity of TP_GS_ lesions, categorized by location.

	SNR Substandard			Yes Percentage				
Location	Yes	No	TP_GS_	95% CI [LL, UL]	*p*			
	*n*	*n*	*n*	*%*				
All	147	421	568	25.9 [22.3, 29.7]				
Frontal	27	163	190	14.2 [9.6, 20.0]	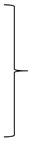	<0.001	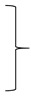	0.07
Parietal	29	91	120	24.2 [16.8, 32.8]				
Temporal	24	111	135	17.8 [11.7, 25.3]				
Occipital	1	17	18	5.6 [0.1, 27.3]				
Central	35	19	54	64.8 [50.6, 77.3]			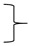	0.67
Infratentorial	31	20	51	60.8 [46.1, 74.2]				
Frontal and Parietal and Temporal and Occipital	81	382	463	17.5 [14.1, 21.3]	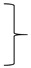	<0.001		
Central and Infratentorial	66	39	105	62.9 [52.9, 72.1]				

Note. SNR = signal-to-noise ratio; TP_GS_ = true-positive lesion counts using the gold standard; CI = confidence interval (Clopper–Pearson); LL = lower limit; UL = upper limit. Brain regions as in Table 11. The *p*-values were calculated using the chi-squared test.

**Table 19:** FLAIR_UF_: CNR assessment in the vicinity of TP_GS_ lesions, categorized by location.

	CNR Substandard			Yes Percentage				
Location	Yes	No	TP_GS_	95% CI [LL, UL]	*p*			
	*n*	*n*	*n*	*%*				
All	33	535	568	5.8 [4.0, 8.1]				
Frontal	6	184	190	3.2 [1.2, 6.8]	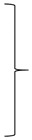	<0.001	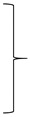	0.15
Parietal	7	113	120	5.8 [2.4, 11.6]				
Temporal	2	133	135	1.5 [0.2, 5.2]				
Occipital	0	18	18	0.0 [0.0, 18.5]				
Central	0	54	54	0.0 [0.0, 6.6]				
Infratentorial	18	33	51	35.3 [22.4, 49.9]				
Frontal and Parietal and Temporal and Occipital and Central	15	502	517	2.9 [1.6, 4.7]	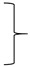	<0.001		
Infratentorial	18	33	51	35.3 [22.4, 49.9]				

Note. CNR = contrast-to-noise ratio; further abbreviations as in Table 18. Brain regions as in Table 11. The *p*-values were calculated using the chi-squared test.

## Data Availability

Anonymized, defaced MRI data can be obtained by the corresponding author upon reasonable request.
